# Lattice softening and diffusive dynamics in the polar metal LiReO_3_

**DOI:** 10.1126/sciadv.adt3886

**Published:** 2026-04-03

**Authors:** Kantaro Murayama, Ryota Masuki, Cédric Tassel, Hideaki Sakai, Tatsuya Yanagisawa, Keito Yoshida, Hiroshi Oike, Suguru Yoshida, Xiangyu Gu, Kohdai Ishida, Morito Namba, Ksenia Denisova, Valérie Dupray, Simon Clevers, Olivier Mentré, Takuya Nomoto, Terumasa Tadano, Craig M. Brown, Peter Lemmens, Ryotaro Arita, Hiroshi Takatsu, Hiroshi Kageyama

**Affiliations:** ^1^ Department of Energy and Hydrocarbon Chemistry, Graduate School of Engineering, Kyoto University, Nishikyo-ku, Kyoto 615-8510, Japan.; ^2^ Department of Applied Physics, The University of Tokyo, 7-3-1 Hongo, Bunkyo-ku, Tokyo 113-8656, Japan.; ^3^ Department of Physics, Osaka University, Toyonaka, Osaka 560-0043, Japan.; ^4^ Department of Physics, Hokkaido University, Sapporo 060-0810, Japan.; ^5^ PRESTO, Japan Science and Technology Agency (JST), Kawaguchi, Saitama 332-0012, Japan.; ^6^ Institute for Condensed Matter Physics, Technische Universität Braunschweig, Braunschweig 38106, Germany.; ^7^ SMS. UR 3233, Univ Rouen Normandie, Normandie Univ, Rouen F-76000, France.; ^8^ Université Lille Nord de France, UMR 8181 CNRS, Unité de Catalyse et de Chimie du Solide (UCCS USTL), Villeneuve d’Ascq F-59655, France.; ^9^ Research Center for Advanced Science and Technology, University of Tokyo, 4-6-1 Komaba, Meguro-ku, Tokyo 153-8904, Japan.; ^10^ Department of Physics, Tokyo Metropolitan University, Hachioji, Tokyo 192-0397, Japan.; ^11^ CMCM, National Institute for Materials Science (NIMS), 1-2-1 Sengen, Tsukuba, Ibaraki 305-0047, Japan.; ^12^ Chemical and Biomolecular Engineering, University of Delaware, Newark, DE 19716, USA.; ^13^ National Institute of Standards and Technology, Center for Neutron Research Gaithersburg, MD 20899-6102, USA.; ^14^ RIKEN Center for Emergent Matter Science, 2-1 Hirosawa, Wako, Saitama 351-0198, Japan.

## Abstract

Polar metals, characterized by the nontrivial coexistence of metallicity and polar structural order, define an emerging frontier in quantum materials research. However, the interplay between their structural phase transitions and fluctuation dynamics remains poorly understood. Here, we reveal distinct diffusive dynamics in metallic lithium rhenium trioxide (LiReO_3_) associated with its polar-to-nonpolar transition. Unlike isostructural lithium niobate (LiOsO_3_) and related systems, LiReO_3_ exhibits pronounced phase fluctuations both above and below *T*_s_. Thermoelectric, Raman, and ultrasound measurements demonstrate a probe-dependent thermal hysteresis, while ultrasound data further show lattice softening and persistent resonant absorption at low temperatures across a broad timescale (1 to 100 microseconds). These observations indicate a multiscale spatiotemporal dynamics governed by a shallow anharmonic potential stabilized by itinerant electrons, as supported by finite-temperature first-principles calculations. By mapping the fluctuation landscape shaped by itinerant electrons, this work offers a previously unexplored perspective for exploiting fluctuation-driven phenomena in polar metals.

## Teaser

Conduction electrons drive fluctuations across shallow energy landscapes in polar metals, persisting at low temperatures.

## INTRODUCTION

Ferroelectric oxides are indispensable for both fundamental studies and technological applications (*1*). Among them, LiNbO_3_ (LN)–type structures are widely used because of their strong piezoelectricity, pyroelectricity, and nonlinear optical properties (*2*, *3*). These materials undergo polar-nonpolar (P-NP) structural phase transitions driven by symmetry breaking (*4*–*6*). Beyond these well-established properties, relaxor ferroelectrics exhibit nanoscale phase inhomogeneity upon cooling, manifested as polar nanoregions (*7*), a phenomenon attributed to the Anderson localization of ferroelectric phonons (*8*). The incorporation of transition metals has further broadened LN-type materials, enabling multiferroicity, as exemplified by MnTiO_3_ (*9*) and FeTiO_3_ (*10*).

In contrast to these insulating materials, polar metals were long considered improbable since their theoretical proposal in the 1960s (*11*), primarily because conduction electrons were expected to screen out polar instabilities and suppress P-NP transitions. This view was overturned five decades later by the discovery of LiOsO_3_, an LN-type compound that undergoes a P-NP transition at *T*_s_ = 140 K while retaining metallicity, thus recognized as the first polar metal undergoing a P-NP structural phase transition (*12*). Since then, the coexistence of polarity and metallicity has attracted growing attention (*13*–*16*). Ultrafast spectroscopy studies suggest that the transition in LiOsO_3_ involves a decoupling between itinerant electrons and transverse optical polar phonons (*17*), whereas high-pressure experiments attribute this decoupling to a local Li-O coordination instability (*18*). Recent second-harmonic generation (SHG) measurements further identified short-range polar correlations persisting above *T*_s_ (*T*_s_ < *T* < ~230 K) (*19*), indicating the presence of a critical region and an order-disorder–type second-order transition, reminiscent of the scenario proposed for LiNbO_3_ (*4*, *5*, *20*–*24*).

Despite these observations, the underlying driving force remains elusive, particularly regarding how the polar metallic state relates to its insulating counterparts. The intricate interplay between P-NP phase transitions and fluctuation dynamics, governed by the unique electronic states of polar metals, remains poorly understood. Furthermore, the functional exploitation of such fluctuations represents an emerging yet largely unexplored direction.

**Fig. 1.**
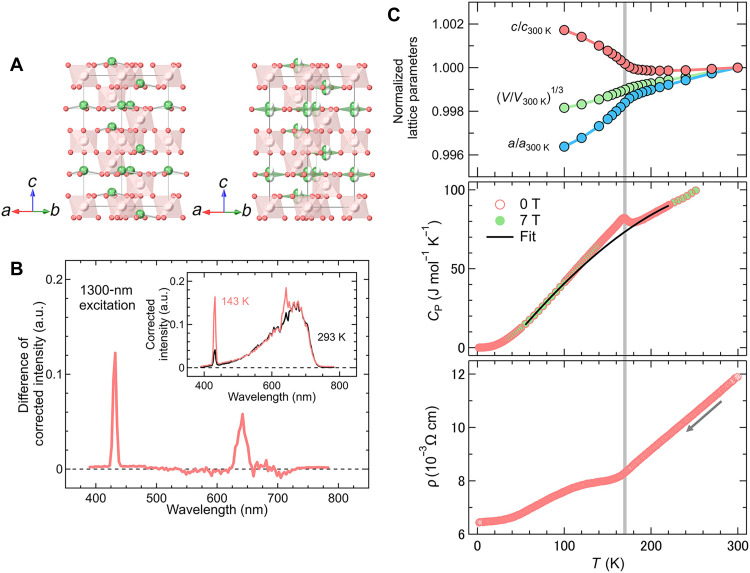
P-NP transition and electronic property of LiReO_3_. (**A**) Crystal structures of LiReO_3_ in the polar (*R*3*c*; left) nonpolar (*R*-3*c*; right) phases. Spheres and polyhedra represent Li^+^ ions and ReO_6_ octahedra, respectively. (**B**) Difference in emission intensity (Δ*I* = *I*_143 K_′ – *I*_293 K_′) as a function of emission wavelength under 1300-nm excitation, highlighting peaks at 650 nm and 433 nm corresponding to second- and third-harmonic generation (SHG and THG), respectively. The corrected intensities *I*_143 K_′ and *I*_293 K_′ were obtained by scaling the data at 143 and 293 K to normalize the fluorescence background in the 400- to 700-nm region, as shown in Fig. 1B (inset; see also the Supplementary Materials). The pronounced SHG signal at 650 nm confirms the noncentrosymmetric structure below *T*_s_ despite strong fluorescence background. a.u., arbitrary units. (**C**) Temperature dependence of (top) lattice parameters normalized to 300 K; (middle) specific heat at 0 T (open) and 7 T (closed), with the black line indicating a fit excluding the transition region; and (bottom) electrical resistivity measured during cooling. All results consistently demonstrate the P-NP transition near 170 K.

In this work, we report that LiReO_3_ is a polar metal undergoing a P-NP transition at a slightly higher temperature (*T*_s_ = 170 K) than LiOsO_3_ (Fig. 1A) but with a fundamentally different character. Unlike LiOsO_3_, which exhibits precursor phenomena only above *T*_s_ (*19*, *25*), LiReO_3_ undergoes a first-order transition marked by diffusive lattice dynamics and persistent spatiotemporal fluctuations that extend well below *T*_s_, accompanied by probe-dependent hysteresis spanning a wide temperature range. The suppression of the polar phase in the insulating-to-metal crossover of LiRe_1–*x*_Nb*_x_*O_3_ highlights the role of mobile electrons in screening the internal electric field. In addition, first-principles calculations reveal an exceptionally small energy difference between polar and nonpolar states, resulting in a shallow potential landscape that stabilizes a biphasic regime over an extended temperature range. These findings provide insights into the interplay between polarity and metallicity, as it enlarges its existence and demonstrates a pathway to accessing and controlling emergent low-temperature functionalities in polar metals.

## RESULTS

### Polar metallic LiReO_3_

Previous structural analyses of LiReO_3_ were limited to room temperature and assumed a polar (*R*3*c*) structure without direct confirmation of polarity (*26*). The authors also noted possible lithium nonstoichiometry or inhomogeneity, potentially resulting from a topochemical reaction from ReO_3_ (*26*). To obtain stoichiometric samples, we used high-pressure synthesis, which effectively suppresses defects commonly present under ambient-pressure conditions (*27*–*29*). The synchrotron x-ray diffraction (SXRD) pattern at 300 K was indexed using a rhombohedral cell [*a* = 5.09984(1) Å, *c* = 13.39810(4) Å] (fig. S1), consistent with previous reports (*26*). However, SHG measurements showed no signal at 293 K, while a clear peak emerged at 143 K (Fig. 1B and fig. S2), indicating the breaking of centrosymmetry below room temperature.

Rietveld refinements of the SXRD data at 300 and 100 K were performed using the centrosymmetric (*R*-3*c*) and noncentrosymmetric (*R*3*c*) space groups, respectively (fig. S1 and tables S1 and S2), with both models yielding regular ReO_6_ octahedra. To further investigate the low-temperature structure, neutron powder diffraction (NPD) at 6 K was conducted, which revealed off-centering of Li^+^ ions (fig. S3 and table S3), as observed in canonical polar oxides such as LiNbO_3_ and LiTaO_3_ (*4*, *30*–*33*). These results collectively confirm the emergence of a polar phase in LiReO_3_ below room temperature, consistent with SHG observations.

In situ SXRD measurements (fig. S4) revealed a P-NP structural phase transition, characterized by anomalous thermal expansion between 160 and 190 K (Fig. 1C, top). This observation is consistent with Raman spectroscopy, which shows additional phonon modes within the same temperature range (160 to 180 K; figs. S5 and S6 and tables S4 and S5). The temperature dependence of the lattice parameters initially suggests a second-order–like transition, further supported by a λ-shaped anomaly in the specific heat at *T*_s_ = 170 K (Fig. 1C, middle). Furthermore, the electrical resistivity ρ exhibits a kink at *T*_s_ while maintaining metallic behavior (dρ/d*T* > 0; Fig. 1C, bottom). These results confirm that LiReO_3_ is a metallic system undergoing a P-NP phase transition at 170 K, a temperature higher than that of LiOsO_3_ (140 K).

### Polar phase instability

The transition temperature (*T*_s_) of LiReO_3_ (170 K) is markedly suppressed to just 12% of that in the isostructural insulating compound LiNbO_3_ (*T*_s_ = 1480 K) (*4*). While this suppression is presumably related to the presence of conduction electrons, the underlying mechanism is unclear. To explore this, we synthesized solid solutions of LiRe_1–*x*_Nb*_x_*O_3_. The SXRD patterns show sharp peaks and a gradual shift in peak positions with increasing *x*, indicating high crystallinity and the successful formation of an entire solid solution (fig. S7).

**Fig. 2.**
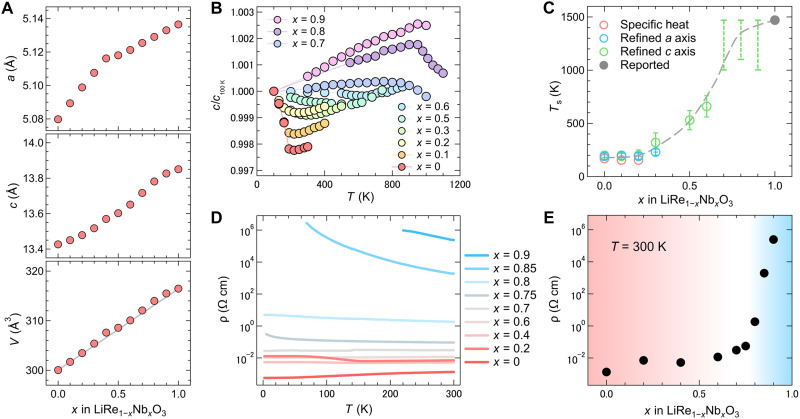
Itinerancy-induced polar phase instability in LiRe_1–*x*_Nb*_x_*O_3_. (**A**) Lattice parameters *a*, *c*, and cell volume *V* at 100 K as a function of Nb content *x* in LiRe_1–*x*_Nb*_x_*O_3_ (0 ≤ *x* ≤ 1). (**B**) Temperature dependence of the *c* axis for selected compositions, normalized at 100 K. (**C**) P-NP phase diagram of LiRe_1–*x*_Nb*_x_*O_3_. Open circles represent *T*_s_ values estimated from lattice parameters (*a*, *c*) and specific heat measurements. The filled circle corresponds to earlier reported data (*4*). Dotted lines at *x* = 0.7, 0.8, and 0.9 represent the possible *T*_s_ region with the maximum measurement temperature as the lower bound and 1470 K (the *T*_s_ of LiNbO_3_) as the upper boundary because no distinct changes appeared in measurements for these compositions up to 1000 to 1100 K. The dashed curve connecting the *T*_s_ values at *x* = 0 and *x* = 1 is a guide to the eye. (**D**) Temperature-dependent resistivity of LiRe_1–*x*_Nb*_x_*O_3_ measured during cooling from 300 to 2 K. (**E**) Room-temperature resistivity as a function of *x*, showing a crossover from itinerant to localized electronic behavior upon electron doping. The (C) and (E) show a notable change near *x* = 0.6 to 0.75 in the polar structure, suggesting the presence of a metal-insulator crossover.

The unit cell volume (*V*) increases linearly with *x*, following Vegard’s law. In contrast, the individual lattice parameters (*a*, *c*) exhibit nonlinear variations (Fig. 2A), likely reflecting electronic effects, as observed in other solid solutions (*34*–*37*). *T*_s_ also evolves nonlinearly: On the Re-rich side, samples with *x* = 0.1 and 0.2 exhibit nearly the same *T*_s_ as the parent compound (*x* = 0), as evidenced by anomalies in the lattice constants (fig. S8) and specific heat (fig. S9). With increasing *x*, *T*_s_ gradually rises and tracks a minimum in the *c*-axis length (Fig. 2B), a trend empirically observed in LiOsO_3_ as well (*12*). For *x*  ≥ 0.7, in situ SXRD measurements up to 1100 K show no signature of a phase transition, suggesting that *T*_s_ continues to increase toward *x* = 1, where it reaches 1480 K in LiNbO_3_ (*4*).

The resulting phase diagram, constructed from structural and specific heat measurements, delineates the evolution from polar metallic to polar insulating phases across the solid solution series (Fig. 2C). While the continuity of the P-NP transition with respect to Nb content *x* remains to be clarified, *T*_s_ shows a strong correlation with the electronic transport properties. As the Nb content decreases from *x* = 1 to 0.75, the resistivity (ρ) drops sharply (Fig. 2, D and E), and the temperature coefficient dρ/d*T* switches from negative to positive, signaling an insulator-to-metal crossover around *x* = 0.7 (Fig. 2, C and E). These observations suggest that the instability of the polar phase originates from a crossover in electronic character, from localized to itinerant electrons, accompanied by enhanced screening of internal electric fields. The nearly flat *T*_s_ region between *x* = 0 and *x* = 0.2 (Fig. 2C) indicates sufficient screening that effectively suppresses variations in *T*_s_. Thus, this systematic investigation of LiRe_1–*x*_Nb*_x_*O_3_ offers clear experimental evidence for the suppression of polar order via conduction-electron screening, reinforcing the pivotal role of itinerancy in destabilizing the polar phase (*11*, *12*).

### Extensive thermal hysteresis of LiReO_3_

**Fig. 3.**
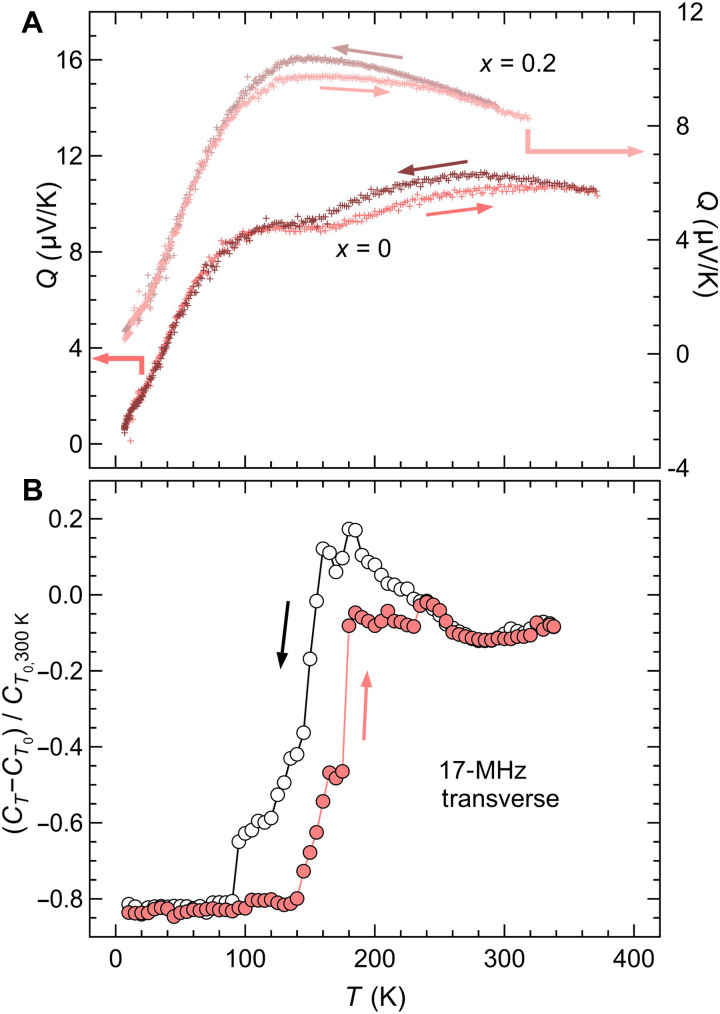
Thermal hysteresis in LiReO_3_. Temperature dependence of (**A**) thermopower *Q* for LiRe_1–*x*_Nb*_x_*O_3_ (*x* = 0, 0.2) determined through thermoelectric measurements and (**B**) transverse elastic constants of LiReO_3_, normalized at 300 K, probed by ultrasonic measurements, showing distinct softening below *T*_s_. The prior increase of the elastic constant above *T*_s_ may arise from reduced phonon anharmonicity (*61*, *100*, *101*) or embryonic domain formation typically observed in first-order phase transitions (*102*–*104*).

As discussed earlier, LiReO_3_ exhibits second-order–like behavior in both specific heat and lattice parameters (Fig. 1C). However, an intriguing feature emerges in its thermodynamic and structural responses: pronounced thermal hysteresis, whose onset temperature and width vary depending on the experimental probe (Fig. 3). Thermoelectric measurements, which are sensitive to thermal fluctuations near P-NP structural instabilities (*38*) and detect direct changes in electronic states near the Fermi level (*S* ∝ ∂*D*/∂*E* |_*E* = *E*F_), reveal weak but extended hysteresis spanning a wide temperature range (100 to 370 K) across *T*_s_ (Fig. 3A). Ultrasonic experiments show marked softening of the transverse elastic constants, with a hysteresis observed between 60 and 240 K (Fig. 3B). These probes, sensitive to quadrupolar and strain susceptibilities, capture lattice dynamics on shorter timescales than those accessed by thermal relaxation processes in specific heat, thus offering complementary insights into electronic and structural fluctuations.

Additional evidence for these exceptional dynamics and fluctuations comes from electrical resistivity and Raman spectroscopy, both of which show persistent hysteresis during heating and cooling cycles (figs. S10 and S11), with signatures extending up to 300 K. The observation of hysteresis in these microscopic or local measurements supports the first-order nature of the P-NP transition in LiReO_3_. While SHG probes the presence of a polar structure, its signal is a time-integrated ensemble of snapshots defined by the ultrafast interaction timescale (~100 fs) and thus cannot distinguish whether the polar structure is static or slowly fluctuating. In contrast, combinations of slower probes such as thermoelectric, ultrasonic, and Raman scattering experiments reveal persistent fluctuations on longer time scales. The variation in hysteresis width among different probes suggests that the underlying dynamics span multiple time and length scales, indicative of microscopic phase coexistence and spatiotemporal fluctuations. This method-dependent behavior is reminiscent of complex dynamical phenomena in other systems, including local instabilities in rattling compounds (e.g., PrOs_4_Sb_12_) (*39*) and spin fluctuations in geometrically frustrated magnets (e.g., NiGa_2_S_4_) (*40*). Given the broad and probe-dependent nature of the hysteresis, determining a single, well-defined transition temperature is not straightforward. In this study, we define *T*_s_ as the temperature at which the specific heat or the *c*-axis lattice parameter reaches a maximum or minimum, thereby characterizing the transition on slower, macroscopic timescales.

### First-principles calculations at finite temperatures

We investigated the phase transitions of metallic LiReO_3_ and insulating LiNbO_3_ using first-principles calculations at finite temperatures. To capture the effects of lattice anharmonicity, we used a recently developed approach based on self-consistent phonon (SCP) theory (*41*–*45*), which explicitly incorporates anharmonic lattice vibrations (*46*, *47*). This method successfully reproduces both the transition temperatures and thermal hysteresis behavior in the two compounds. The calculated transition temperatures, *T*_s,calc_ = 267 K for LiReO_3_ and 1350 K for LiNbO_3_, are in reasonable agreement with the experimental values (170 and 1480 K, respectively) (*4*), demonstrating that our model effectively captures the essential features of the P-NP transitions.

**Fig. 4.**
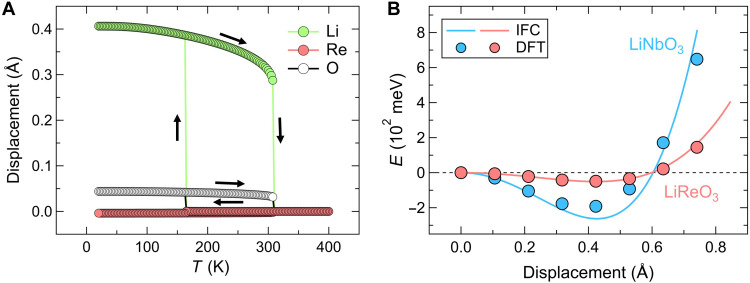
Theoretical calculations for metallic LiReO_3_ and insulating LiNbO_3_. (**A**) Calculated atomic displacements in LiReO_3_ as a function of temperature based on interatomic force constants (IFCs), showing a dispersive hysteresis when only the long-range order is considered. (**B**) Calculated energy per unit cell for LiRe_1–*x*_Nb*_x_*O_3_ (*x* = 0, 1) as a function of the polar mode amplitude, normalized to the Li displacement. For both LiReO_3_ and LiNbO_3_, the circles and lines represent results from density functional theory (DFT)– and IFC-based calculations, respectively.

The calculations reveal a notable contrast in thermal hysteresis. LiReO_3_ exhibits an extended hysteresis window of Δ*T*_calc_ = 145 K (165 to 310 K; Fig. 4A), while LiNbO_3_ shows a much narrower hysteresis of ~40 K (fig. S12). This difference stems from their distinct potential energy landscapes (Fig. 4B): LiNbO_3_ has a deep potential well, resulting in a high transition temperature and minimal hysteresis. In contrast, LiReO_3_ exhibits a shallow double-well potential that amplifies thermal fluctuations and facilitates phase competition over a broad temperature range. These features are qualitatively consistent with experimental observations, namely, a lower *T*_s_ and broader hysteresis in LiReO_3_, suggesting that the shallow potential, likely stabilized by conduction electrons, plays a central role in its first-order–like transition behavior. While SCP calculations also predict a first-order phase transition for LiOsO_3_ (*T*_s_ = 207 K, Δ*T*_calc_ = 120 K), experimental studies (*12*, *19*, *25*, *48*–*50*) report a continuous, second-order phase transition without any detectable signatures of hysteresis (*12*, *19*, *25*, *48*–*50*).

Although shallow potential wells (~50 meV) are also present in conventional insulating ferroelectrics such as BaTiO_3_ (*51*, *52*), they do not give rise to the extended thermal hysteresis observed in LiReO_3_ (*53*), highlighting the critical role of electron itinerancy. Moreover, experimental signatures such as two-phase coexistence and short-range fluctuations in LiReO_3_ point to complexities that are inherently difficult to capture within current first-principles frameworks, posing a substantial challenge for further theoretical developments.

### Fluctuations in the low-temperature regime

Unlike LiOsO_3_, LiReO_3_ exhibits persistent and unconventional fluctuations well below *T*_s_. A clear macroscopic manifestation appears in the electrical resistivity, which displays a broad hump below *T*_s_ (Fig. 1B). This behavior contrasts with the valley-like drop observed in LiOsO_3_ (*12*), which is characteristic of coherent long-range ordering in a Fermi liquid state (*54*–*56*). On a microscopic scale, the Raman spectrum of LiReO_3_ at 4 K remains unusually broad and diffuses over a wide frequency range (fig. S6). This spectral profile suggests that dynamic fluctuations persist on the timescale of optical phonons, indicative of an unresolved P-NP state. This behavior resembles quasielastic scattering associated with correlated or cooperative disorder, as reported in martensitic precursors (*57*), ice-rule systems (*58*), and mixed-anion compounds (*59*). Additional evidence comes from elastic measurements: The transverse elastic constants exhibit continuous softening well below *T*_s_ (Fig. 3B), deviating from conventional structural transitions where softening typically occurs near the critical point and is followed by rapid hardening (*60*). This anomaly highlights an unusual mechanical “softness” in the low-temperature phase, likely associated with spatial modulations due to the proximity of competing polar and nonpolar states.

**Fig. 5.**
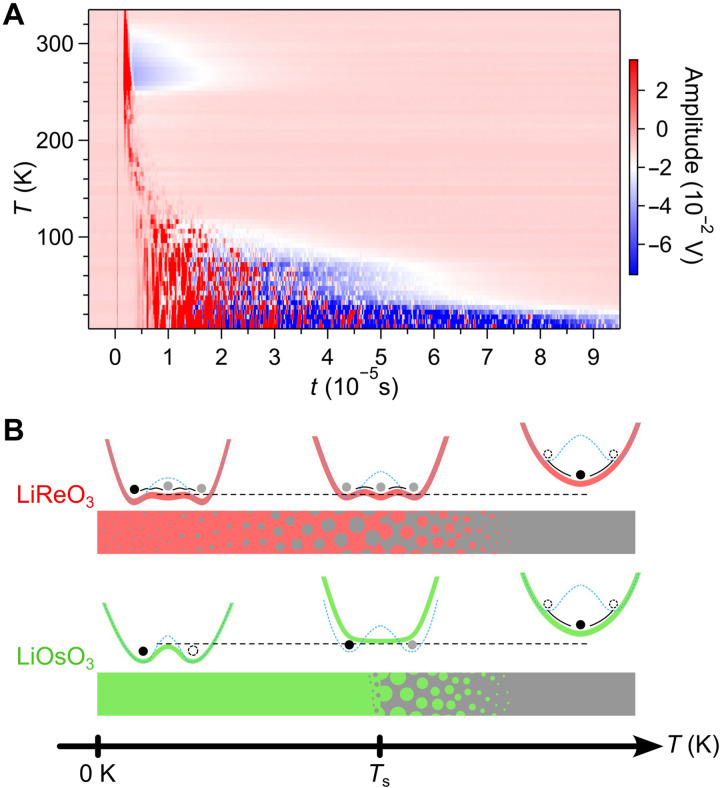
Dynamic fluctuations in polar metallic LiReO_3_. (**A**) Temperature-dependent ultrasonic echo profiles of LiReO_3_ from 10 to 335 K, collected upon heating using a 17-MHz transverse wave. (**B**) Schematic illustration of the P-NP transition in LiReO_3_ and LiOsO_3_, depicting potential curves for the first-order transition in LiReO_3_ versus the second-order transition in LiOsO_3_. The shallow potential in LiReO_3_, attributed to screening effects of conduction electrons (as indicated by theoretical calculations in Fig. 4), facilitates phase fluctuations. These fluctuations are schematically depicted in a bar diagram with red (polar) and gray (nonpolar) regions illustrating their temperature evolution. LiOsO_3_ undergoes a second-order phase transition likely influenced by more pronounced Li thermal disorder (represented by dashed lines; discussed in detail in the main text), with its corresponding bar diagram showing fluctuations present only in the critical region around and above *T*_s_.

A central highlight of this study arises from ultrasonic echo measurements. Echo signals spanning a wide temporal range (1 to 100 μs; Fig. 5A) reveal exceptionally slow and complex spatiotemporal fluctuations that persist down to 10 K, well below *T*_s_. Unlike typical structural transitions, where ultrasonic absorption diminishes because of reduced anharmonic phonon scattering (*61*), LiReO_3_ exhibits anomalous absorption behavior, suggesting that acoustic waves remain resonantly coupled to the polar phase or fluctuations. This behavior indicates a diffusive and dynamically fluctuating lattice state, driven by the small energy separation between the polar and nonpolar phases. These results underscore a strong coupling between acoustic and electronic degrees of freedom mediated by phonon-electron interactions, presenting a compelling case of itinerancy-enhanced lattice dynamics. Moreover, they suggest that such fluctuations in soft polar metals may serve as memory elements, enabling spatially localized and temporally delayed electrical responses to acoustic stimuli.

Together, the probe-dependent hysteresis, broad Raman features, and anomalous ultrasonic responses indicate a highly diffusive lattice environment, consistent with a first-order phase transition accompanied by persistent fluctuations well below *T*_s_. These findings establish LiReO_3_ as a rare polar metal that exhibits multiscale dynamical behavior, bridging characteristics of both displacive and order-disorder transitions.

## DISCUSSION

### Phase transition mechanisms in LiReO_3_ and LiOsO_3_

Despite having identical crystal structures and itinerant electronic states, LiReO_3_ and LiOsO_3_ undergo fundamentally different P-NP transitions: first-order in LiReO_3_ and second-order in LiOsO_3_ (*12*, *19*, *25*, *48*–*50*). This contrast is not captured by the current theoretical models (see above), suggesting that additional effects, such as short-range ordering and thermal disorder of Li displacements, play essential roles beyond current approximations. Raman scattering experiments reveal that LiReO_3_ exhibits softened phonon modes in the 340- to 400-cm^−1^ range compared to LiOsO_3_ (table S4 and4 fig. S5) (*48*–*50*). Density functional theory (DFT) calculations show that these modes correspond to octahedral vibrations involving shear displacements of Li ions, in contrast to the axial Li motion typically observed in polar transitions of LN-type compounds. In LiReO_3_, the coupling between these soft shear modes and the polar-active mode likely stabilizes a collective lattice distortion, suppressing Li disorder and promoting a first-order phase transition.

Recent SHG measurements on LiOsO_3_ have revealed a polar phase with short-range correlations within the nonpolar phase (*T*_s_ < *T* < ~230 K) (*19*), indicating a critical region and suggesting an order-disorder–type second-order transition (Fig. 5B, bottom), similar to the scenario proposed for LiNbO_3_, although the mechanism remains under debate (*4*–*6*, *20*–*24*, *62*, *63*). The Raman frequency of the Li shear mode in LiNbO_3_, responsible for coupling with the polar-active axial Li vibrations, closely matches that in LiOsO_3_ (*24*, *64*, *65*). This fact implies that Li thermal disorder plays a similar role in those compounds. In contrast, the softer Li shear mode in LiReO_3_ likely acts to suppress such disorder, resulting in a first-order transition primarily driven by a displacive-type mechanism (Fig. 5B, top). This soft shear mode may also amplify phase fluctuations through further shallowing of the potential energy surface, reinforcing the first-order nature of the transition.

Figure 5B schematically illustrates these contrasting phase transition pathways in LiReO_3_ and LiOsO_3_. In LiOsO_3_, strong Li order-disorder effects dominate, leading to second-order behavior (Fig. 5B, bottom). In LiReO_3_, these effects are less pronounced, allowing a first-order transition to emerge, stabilized by a shallow potential energy landscape (Fig. 5B, top). The nature of P-NP phase transitions in LiNbO_3_-type compounds has long been debated, particularly regarding the competition between displacive and order-disorder mechanisms. Our results provide what is likely the first compelling experimental evidence of a first-order transition within this class of materials.

The slightly higher *T*_s_ in LiReO_3_ (170 K) compared to LiOsO_3_ (140 K) also merits serious consideration. This difference likely reflects variations in their d-electron counts: The higher d-electron occupancy in LiOsO_3_ enhances conduction electron screening, which suppresses polar distortions and lowers *T*_s_. However, transition temperatures are ultimately governed by a complex interplay between electronic and lattice degrees of freedom, including interatomic force constants (IFCs), and remain an active topic of investigation.

### Final remarks and outlook

Fluctuations above the critical temperature (*T*_c_) are observed across a wide range of materials and are often characterized by inhomogeneous nanoregions (*66*). These include pseudogaps and electronic nematic phases in unconventional superconductors (*67*–*69*), as well as pretransitional behavior in martensitic transformations of intermetallic compounds (*57*, *70*–*73*) and oxides such as LaNbO_4_ (*74*) and Pb_3_(PO_4_)_2_ (*75*), where nucleation and growth processes are mediated by “ghost lattices” or “embryonic fluctuations.” Similar phenomena occur in ferroelectric BaTiO_3_ (*76*–*82*) and in first-order electronic transitions in Mott insulators and charge-ordered systems (*83*–*89*).

While most studies focus on phase fluctuations above transition temperatures, our work on LiReO_3_ reveals a rare case in which a first-order phase transition coexists with persistent dynamical fluctuations both above and below *T*_s_. This unusual behavior, driven by a shallow energy landscape and facilitated by electron itinerancy in conjunction with the P-NP transition, represents a major advance in our understanding of fluctuating states in quantum materials. Unlike conventional systems that respond uniformly to external fields, materials with inherent fluctuations may exhibit nonlinear, delayed, or selective responses to stimuli such as heat, light, or acoustic perturbations (*90*–*92*).

This insight advances the emerging concept of “fluctuation-based materials science,” which recognizes dynamic structural and electronic fluctuations as valuable resources rather than defects to be eliminated. These fluctuations can drive emergent spin and electronic functionalities while producing nonlinear responses that remain unattainable in statically ordered systems. By repositioning fluctuations from sources of noise to active design parameters and harnessing external stimuli such as ultrasound as control mechanisms, promising opportunities emerge for energy conversion and adaptive electronic technologies. The unique dynamical behavior observed in LiReO_3_ thus provides a compelling platform for exploring fluctuation evolution in polar metals and for developing materials design principles that leverage dynamic degree of freedom to achieve emergent electronic and structural functionalities.

## MATERIALS AND METHODS

### Sample preparation

Polycrystalline samples of LiRe_1–*x*_Nb*_x_*O_3_ (0 ≤ *x* ≤ 1) were synthesized via high-pressure and high-temperature solid-state reactions using stoichiometric NbO_2_ (Rare Metallic; 99.9%), ReO_2_ and a 20% molar excess of Li_2_O_2_ (Sigma-Aldrich; 90%). ReO_2_ powder was prepared using Re (Sigma-Aldrich; 99.995%) and Re_2_O_7_ (Sigma-Aldrich; 99.99%) in an evacuated silica tube ~ 5 Pa at 300°C and then 650°C for 1 day each at a heating rate of 200°C hour^−1^. The chemicals for high-pressure reactions were ground, pelletized, and wrapped in platinum foils of 0.02-mm thickness and inserted into boron nitride (BN) sleeves. After sealing both ends of the sleeve with BN lids, the sample cell was loaded into a graphite tube and introduced into pyrophyllites. These assembly procedures were conducted in an N_2_-filled glovebox due to the air-sensitive precursors. After pressurization to reach 8 to 9 GPa, the samples were heated to 1100° to 1400°C for 1 hour and quenched down to room temperature within 5 min, and then the pressure was slowly released to the ambient atmosphere. The pellets were crushed into powders and washed with water to remove residual lithium-related by-products and unreacted precursors. As-prepared pellets were used for resistivity, thermoelectric, and ultrasonic measurements.

### Characterization of samples

SXRD experiments for LiRe_1–*x*_Nb*_x_*O_3_ were performed at the BL02B2 beamline in SPring-8. The wavelength of the incident beam was λ = 0.420150 and 0.420391 Å, and the temperature was changed in the range from 100 to 1100 K. NPD measurements of LiReO_3_ (*x* = 0) were performed at 6 K using the high-resolution powder diffractometer BT-1 at the National Institute of Standards and Technology (NIST) Center for Neutron Research. Incident neutrons of wavelength λ = 1.5400 Å monochromated by vertical-focused Cu(311) monochromator were used. These diffraction data were analyzed by the JANA2006 software (*93*). SHG measurements were performed using a TCS SP8 MP confocal microscope (Leica Microsystems, Wetzlar, Germany) coupled to a tunable InSight X3 single laser (Spectra-Physics, USA) emitting femtosecond pulse (100 fs/80 MHz). For this experiment, an excitation wavelength of 1300 nm for a laser power of 0.25 W was chosen. The sample was placed in a computer-controlled heating/cooling state (Linkam THMS600) and cooled between 293 and 143 K at a cooling rate of 5 K min^−1^. The SHG intensity was measured as digital counts (0 to 255 levels) from the multiphoton microscope detectors, averaged over all pixels in the acquired image. To clarify the temperature-dependent component associated with SHG, the spectrum at 293 K was subtracted from the low temperature spectrum at 143 K after appropriate scaling (see the Supplementary Materials). The acquisitions of the spectral emission of the sample were performed between 380 and 780 nm.

### Physical property measurements

Specific heat measurements were carried out using a commercial calorimeter (Quantum Design, PPMS) with a thermal relaxation method. Entropy changes near the phase transition were also determined through specific heat measurements (fig. S13). Resistivity measurements were performed using the physical property measurement system (Quantum Design, PPMS). Magnetic susceptibility (*M*/*H*) was measured with a SQUID magnetometer (Quantum Design, MPMS; fig. S14). Thermoelectric measurements were performed by a steady-state method with a temperature difference (Δ*T*) of less than 1 K (typically 2 to 4% of the measurement temperature below 50 K) between the voltage contacts. Raman scattering experiments were performed with a HORIBA Jobin Yvon model HR800 micro Raman spectrometer using a 50× objective. The spectra were collected in quasi-backscattering geometry using a λ = 532 nm laser line of a Nd:YAG laser with a power of *P* = 0.4 mW. Low-temperature data were sampled with single crystals inserted in a CRYOVAC, He-cooled micro-cryostat in the temperature range between 4 and 300 K. The energy range of the experiments is 20 to 500 cm^−1^. Ultrasound measurements were conducted on as-synthesized LiReO_3_ pellets in a temperature range from 2 to 335 K and in magnetic fields at 0 T. To generate and detect ultrasonic waves, piezoelectric plates of LiNbO_3_ transducers were attached to the samples using epoxy glue.

### Theoretical calculations

The electronic state of polar LiReO_3_ was calculated using the projected-augmented plane-wave (PAW) method implemented in the Quantum ESPRESSO package (*94*–*96*). We used Perdew-Burke-Ernzerhof functional of generalized gradient approximation as an exchange-correlation functional. The calculations used pseudopotentials Li.rel-pbe-s-kjpaw_psl.1.0.0.UPF, Re.pbe-spn-kjpaw_psl.1.0.0.UPF, and O.pbe-n-kjpaw_psl.1.0.0.UPF. Because the used Li pseudopotential is “hard” and requires a high cutoff energy (suggested minimum: 103 Ry) for precision, we used the cutoff energy of 120 Ry. The *k*-point mesh was set to 14 by 14 by 14, and the Gaussian smearing was used with width of 0.003 Ry. The total energy was minimized until the convergences fell to less than 10^−7^ eV during self-consistent cycles, and the lattice relaxations were conducted until the atomic forces became smaller than 0.02 eV Å^−1^. Density of states (DOS) (fig. S15) were evaluated without Hubbard *U* correction.

The calculations of the P-NP transition were performed using the finite-temperature structural optimization based on SCP theory (*46*, *47*). The temperature dependence of the crystal structure was calculated by optimizing the structure-dependent free energy, which was obtained using the SCP theory. SCP theory is a commonly used method for quantitative calculations of strongly anharmonic materials because the phonon anharmonicity is nonperturbatively treated (*41*–*45*). The SCP calculation and the finite-temperature structural optimizations were performed using ALAMODE package (*42*, *97*, *98*). We used the Vienna Ab initio Simulation Package (*99*) for the DFT calculations. In calculating the temperature dependence of the crystal structure, we fixed the shape of the unit cell because the lattice constants showed only small changes (around 0.2%) below and above *T*_s_. The anharmonic IFCs, which we truncated at the fourth-order, were obtained using the compressive sensing method from the displacement-force data. For more details on the theoretical calculations, including DFT-based phonon calculations for Raman scattering analysis and Gibbs free energy (fig. S16), see “Details of theoretical calculations” in the Supplementary Materials.

Certain commercial equipment, instruments, or materials are identified in this document. This identification does not imply recommendation or endorsement by the NIST or does it imply that the products identified are necessarily the best available for the purpose.

## Supplementary Materials

### This PDF file includes:

Supplementary Text Figs. S1 to S16 Tables S1 to S5 References
