# Supplementary material for: Lattice softening and diffusive dynamics in the polar metal LiReO3
Source: Sci Adv. 2026 Apr 3;12(14):eadt3886. doi: 10.1126/sciadv.adt3886 (PMC13048238; doi:10.1126/sciadv.adt3886)
Supplement: Supplementary file 1 — Supplementary Text Figs. S1 to S16 Tables S1 to S5 References [file sciadv.adt3886_sm.pdf]

Science Advances
AAAS

# Supplementary Materials for

## Lattice softening and diffusive dynamics in the polar metal $LiReO_3$

Kantaro Murayama *et al.*

Corresponding author: Ryotaro Arita, arita@riken.jp; Hiroshi Takatsu, takatsu@scl.kyoto-u.ac.jp; Hiroshi Kageyama, kage@scl.kyoto-u.ac.jp

*Sci. Adv.* **12**, eadt3886 (2026)
DOI: 10.1126/sciadv.adt3886

**This PDF file includes:**

Supplementary Text
Figs. S1 to S16
Tables S1 to S5
References

## Supplementary Text

Rietveld refinements: synchrotron X-ray diffraction of $LiReO_3$

The SXRD patterns of $LiReO_3$ obtained at 300 K and 100 K can be fitted with rhombohedral cells. Rietveld refinements of 300 K and 100 K patterns were conducted using centrosymmetric $R\overline{3}c$ and non-centrosymmetric $R3c$ space groups, respectively. The trends in the lattice parameters of $LiTaO_3$ ($a$ = 5.15428(1) Å, $c$ = 13.78351(2) Å where values in parentheses indicate $\pm 1\sigma$) (*32, 33*), $LiOsO_3$ ($a$ = 5.06379(5) Å, $c$ = 13.2110(2) Å) (*12*) and $LiReO_3$ ($a$ = 5.09983(1) Å, $c$ = 13.39809(4) Å) at 300 K are consistent with the ionic radius of the B-site ($Ta^{5+}$: 0.64 Å, $Os^{5+}$: 0.545 Å, $Re^{5+}$: 0.58 Å) (*105*). In metallic $LiReO_3$, as in $LiOsO_3$, the displacement of Re at the B site is smaller than that of Nb and Ta in insulating $LiNbO_3$ and $LiTaO_3$ (*30 – 33*).

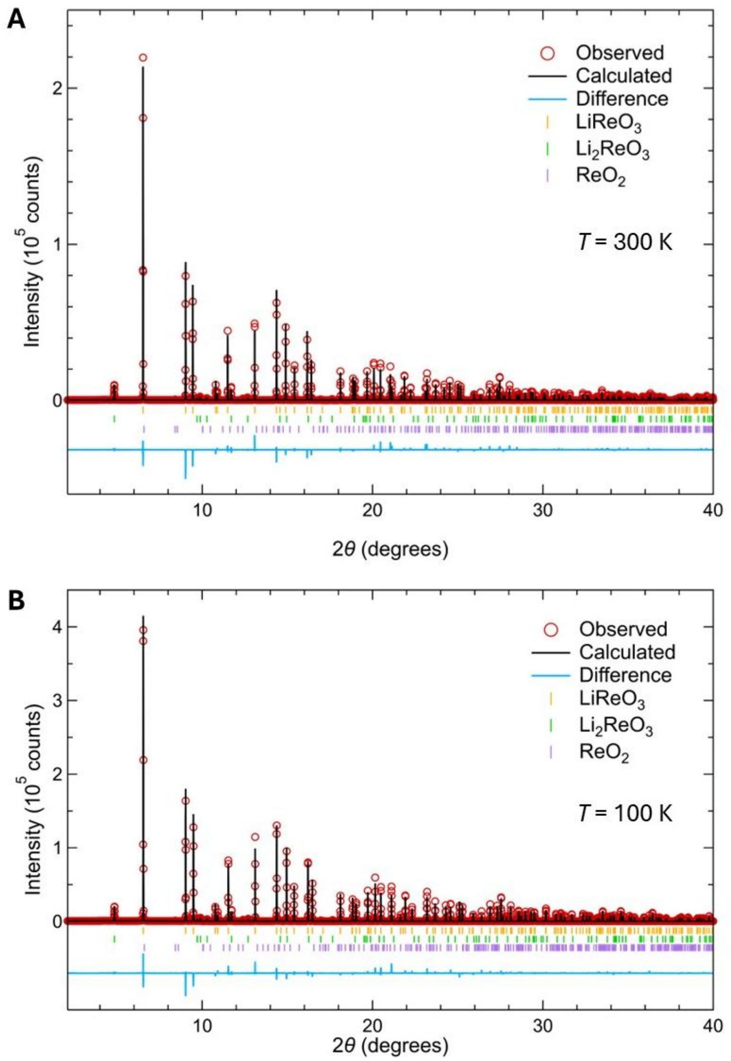


**Fig. S1.** SXRD Rietveld refinement of $LiReO_3$ collected at (a) 300 K and (b) 100 K. The wavelength was 0.420150 Å. The patterns of (a) and (b) were refined using a nonpolar $\boldsymbol{R\overline{3}c}$ and polar $R3c$ space groups, respectively. For both figures, red circles, black and blue lines show observed, calculated and differences, respectively. Orange, green and purple bars show the Bragg peaks of $LiReO_3$, $Li_2ReO_3$ and $ReO_2$, respectively.

**Table S1.**

Structural parameters of $LiReO_3$ determined from SXRD data at 300 K, refined in the $R\overline{3}c$ space group. The occupancy parameters $g$ for each ion were fixed during the refinement. The $z$ parameter of the Li position was fixed at the value determined from NPD experiments (table S3). Analysis using the ideal (undistorted) position of $z$ = 0.25 showed negligible differences. Values in parentheses indicate ±1σ.

| Atom | Site | $x$ | $y$ | $z$ | $g$ | $U_{iso}$ (100 Å$^2$) | $U_{11}$ (100 Å$^2$) | $U_{33}$ (100 Å$^2$) |
|---|---|---|---|---|---|---|---|---|
| Li | 6*a* | 0 | 0 | 0.2826 | 0.5 | 1.2 | | |
| Re | 6*a* | 0 | 0 | 0 | 1 | | 0.631(1) | 0.338 (3) |
| O | 18*b* | 0.6140(1) | 0 | 0.25 | 1 | 1.58 (2) | | |

Space group: $R\overline{3}c$, $a$ = 5.09983(1) Å, $c$ = 13.39809(4) Å, $V$ = 301.776(1) Å$^3$, $R_p$ = 9.90%, $R_{wp}$ = 13.56%.

**Table S2.**
Structural parameters of $LiReO_3$ determined from SXRD data at 100 K, refined in the *R*3*c* space group. The occupancy parameters *g* for each ion were fixed during the refinement. The *z* parameter of Li position was fixed at the value determined from NPD experiments (table S3). Analysis using the ideal (undistorted) position of $z = 0.25$ showed negligible differences.

| Atom | Site | *x* | *y* | *z* | *g* | $U_{iso}$ (100 Å$^2$) | $U_{11}$ (100 Å$^2$) | $U_{33}$ (100 Å$^2$) |
|---|---|---|---|---|---|---|---|---|
| Li | 6*a* | 0 | 0 | 0.2826 | 1 | 1.2 | | |
| Re | 6*a* | 0 | 0 | 0 | 1 | | 0.0964(9) | 0.018(2) |
| O | 18*b* | 0.60(4) | –0.0084(7) | 0.2549(2) | 1 | 0.38(2) | | |

Space group: *R*3*c*, $a = 5.079760(9)$ Å, $c = 13.42621(3)$ Å, $V = 300.0340(9)$ Å$^3$, $R_p = 8.70\%$, $R_{wp} = 11.57\%$.

SHG emission spectra of $LiReO_3$

SHG measurements at 293 K showed broad fluorescence spectra across 500 – 700 nm, which serves as a background signal (Fig. 1B inset, fig. S2). At a lower temperature of 143 K, however, a distinct SHG response at 650 nm emerged over this background, confirming spatial inversion symmetry breaking upon phase transition. Overall emission intensity at 143 K decreased relative to 293 K, likely due to multiple factors, including temperature-induced quenching of fluorescence background in the 400 – 700 nm region. Despite this intensity reduction, the overall spectral shapes of fluorescence background remain~~s~~ nearly identical at both temperatures.

We therefore treated the fluorescence in the 400 – 700 nm region as background and applied multiplicative correction to both datasets at 143 K and 293 K to match their behavior in this region before calculating their difference. This extracts the emergence of SHG (650 nm) and enhancement of THG (433 nm) signals accompanying the phase transition.

Figure S2(b) and (c) show the corrected data ($I_{143K}'$ and $I_{293K}'$) and their difference $\Delta I = I_{143K}' - I_{293K}'$. Here, $I_{293K}'$ was adjusted by scaling $I_{293K}$ to match $I_{143K}$ in the 400 – 700 nm fluorescence range. A scaling factor $\alpha = 0.3233(2)$ was determined by least-squares fitting, yielding $I_{293K}' = \alpha I_{293K}$ and $I_{143K}' = I_{143K}$. Near-zero $\Delta I$ outside SHG/THG regions confirms appropriate correction. The pronounced SHG response at 650 nm provides strong evidence for inversion symmetry breaking following the phase transition below 293 K. Meanwhile, the enhanced THG intensity at 433 nm reflects changes in the nonlinear optical susceptibility $\chi^{(3)}$ owing to symmetry breaking, electronic reorganization, and structural modification during the transition, phenomena known to alter THG responses (*106–110*). The simultaneous enhancement of the THG thus offers strong, complementary evidence of the phase transition.

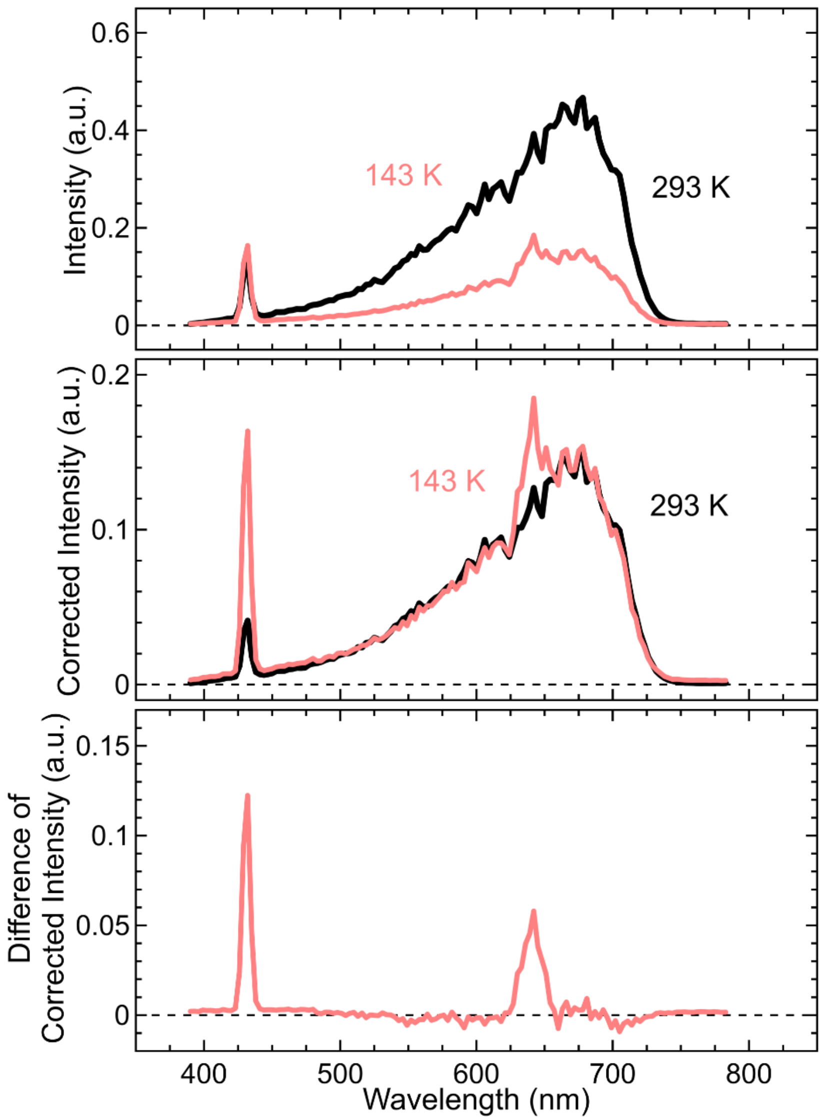


**Fig. S2**. (a) Raw data of emission intensity from SHG measurements at 293 K and 143 K. (b) Corrected emission intensity at 293 K and 143 K and (c) their difference *ΔI*. Data were corrected to match the fluorescence background in the 400 – 700 nm region (see text for more details).

Rietveld refinements: neutron powder diffraction of $LiReO_3$

The NPD pattern of $LiReO_3$ obtained at 6 K can be fitted with rhombohedral cells. Rietveld refinement pattern was conducted using non-centrosymmetric *R*3*c* space groups. We detected lithium ion off-centering consistent with values reported in $LiNbO_3$ and $LiTaO_3$ (*30–33*). Combined with SHG results, this confirms $LiReO_3$ has a polar structure at low temperatures. NPD measurements showed no additional peaks, indicating an absence of magnetic ordering.

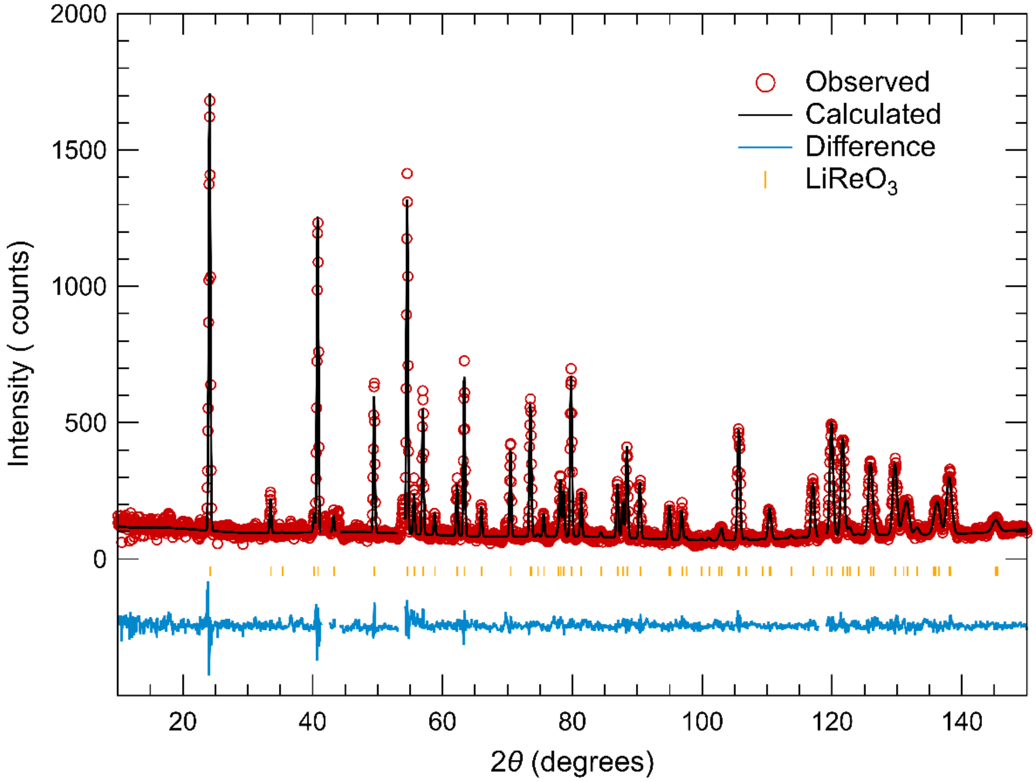


**Fig. S3.** NPD Rietveld refinement of $LiReO_3$ collected at 6 K. The NPD pattern was refined using a polar *R*3*c* space group. No additional peaks indicative of magnetic phase transitions were detected at 6 K, consistent with magnetic susceptibility measurements that showed no anomalies between 2 and 350 K (fig. S14).

**Table S3.**
Structural parameters of $LiReO_3$ determined from NPD data at 6 K, refined in the *R*3*c* space group.

| Atom | Site | *x* | *y* | *z* | *g* | $U_{iso}$ (100 Å$^2$) |
|---|---|---|---|---|---|---|
| Li | 6*a* | 0 | 0 | 0.2826(10) | 1 | 1.2(3) |
| Re | 6*a* | 0 | 0 | 0 | 1 | 0.3(5) |
| O | 18*b* | 0.0524(1) | 0.340(2) | 0.2549(2) | 1 | 0.53(5) |

Space group: *R*3*c*, *a* = 5.07615(8) Å, *c* = 13.4240(4) Å, $R_p$ = 10.49%, $R_{wp}$ = 13.23%.

Temperature-dependent SXRD patterns of $LiReO_3$

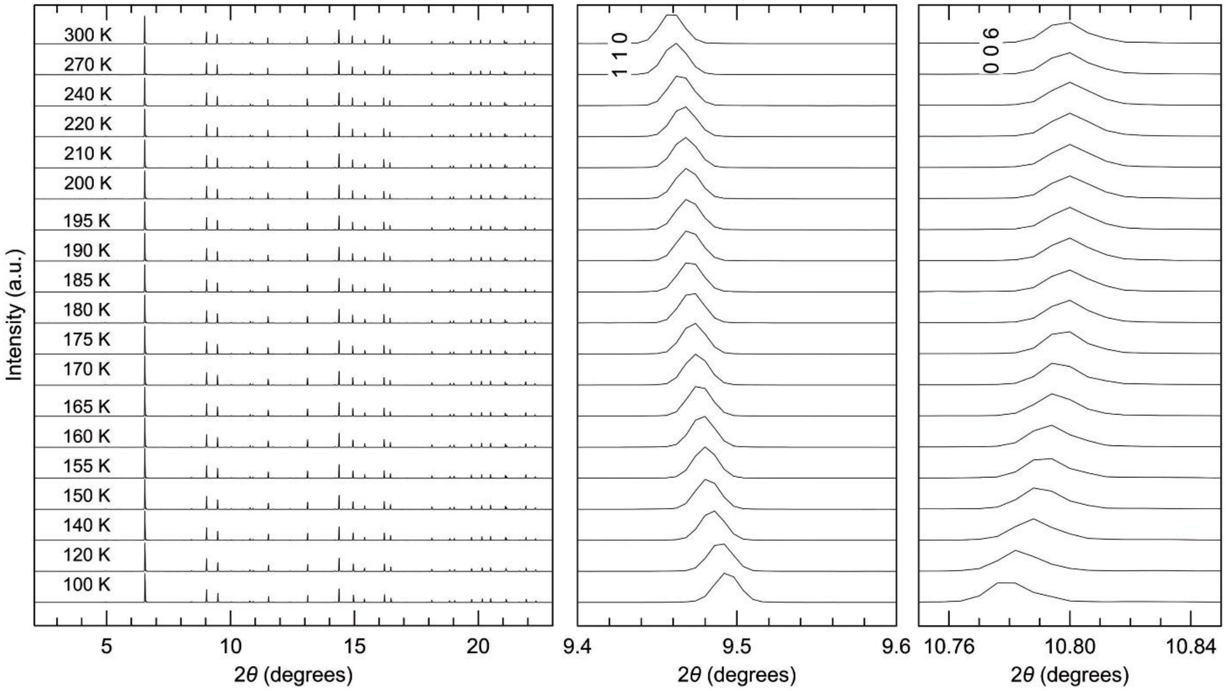


**Fig. S4.** SXRD plots of $LiReO_3$ as a function of temperature from 300 K to 100 K, collected on cooling. The wavelength of the incident beam was $\lambda$ = 0.420391 Å. (left) The overview of the SXRD patterns. (center) Elongated 110 peak showing the expansion along the *ab*-plane. (right) Elongated 006 peak which shows the lattice evolution along the *c*-axis. Uniaxial negative thermal expansion of the *c*-axis below $T_s$ also has been reported for isostructural polar metal $LiOsO_3$ (*12*).

Detailed analysis of Raman scattering spectra of $LiReO_3$

While the main text discusses Raman scattering results for $LiReO_3$, here we present detailed experimental results alongside DFT-based peak analysis and thorough comparisons with $LiOsO_3$. Raman scattering measurements from 300 K to 4 K (fig. S5) revealed additional spectral features between 180 K and 160 K, indicating a phase transition within this temperature range. We analyzed experimental spectra using DFT calculations to assign the Raman active vibrational modes, with computational details provided in the section "Details of theoretical calculations" below. For comparative analysis, we performed similar calculations on $LiOsO_3$ following previous results (*48 – 50*) and compared them with $LiReO_3$ (table S4). The calculations showed good agreement with all experimental peaks of Raman-active modes in the $R$–3$c$ structure of $LiReO_3$ above the transition temperature and accounted for most peaks observed below the phase transition (table S5, fig. S5). Two peaks at 442 $cm^{-1}$ and 482 $cm^{-1}$, observed below the phase transition, could not be reproduced in our calculations. These peaks likely correspond to longitudinal optical (LO) modes, which occur at higher frequencies than transverse optical (TO) modes. In similar materials $LiNbO_3$ and $LiTaO_3$, LO modes appear 20 – 200 $cm^{-1}$ above TO modes (*24*, *64*, *65*). The LO/TO mode splitting arises from interactions between ionic charges and macroscopic electric fields (*24*), which are not included in our current calculations. Alternative scenarios such as multi-phonon scattering processes (*111*, *112*) and phonon confinement (*113*) remain possible, and further experimental studies using single crystals, along with more detailed calculations, are necessary to clarify this aspect.

Comparing $LiReO_3$ and $LiOsO_3$, Raman scattering measurements show that peaks of $LiReO_3$ shift to lower energies (softening or redshift) than those of $LiOsO_3$ (table S4, fig. S5). This softening is most pronounced in peaks between (340 – 400) $cm^{-1}$, which arise from octahedral modes where lithium ions exhibit shear motions perpendicular to the polar axis below the phase transition temperature. The differences in Raman peak intensities between $LiReO_3$ and $LiOsO_3$ is likely attributable to intrinsic factors such as the selection rules, scattering volumes, and electronic polarizability of Re and Os compounds. These properties directly influence the observed phonon mode intensities.

A key distinction in their Raman spectra at low temperature is that $LiOsO_3$ exhibits sharp, well-defined peaks, whereas $LiReO_3$ presents broader peaks and a diffuse spectral intensity over a wider frequency range (fig. S6). This broadening in $LiReO_3$ likely results from substantial dynamic fluctuations, as Raman scattering averages information over the optical coherence length scale. Consistent with this, ultrasonic experiments reveal resonant absorption persisting even at 10 K (Fig. 5A of the main text), suggesting that $LiReO_3$ remains in a fluctuating state on the timescale of Raman measurements ($10^{-13}$ to $10^{-12}$ seconds). In contrast, $LiOsO_3$ undergoes a static phase transition without thermal hysteresis, leading to sharper spectral features.

The diffuse spectral intensity in $LiReO_3$ closely resembles quasi-elastic scattering spectra observed in the precursor regions of martensitic transformations (*57*), ice and spin ice systems (*58*), and mixed anion compounds with correlated disorder (*59*). The evolution of such diffuse scattering has attracted significant scientific interest as a distinctive signature of systems exhibiting "correlated" or "cooperative" disorder and associated fluctuations (*57–59*). It is also worth noting that peak broadening and downshifted Raman frequencies are observed, associated with phonon confinement arising from structural miniaturization and spatial constraints, as demonstrated in studies on nanomaterials such as nanoporous silicon (*113*) and nanodiamond (*114*). This implies that the relatively small-scale spatial fluctuations present in $LiReO_3$ may also contribute to the observed Raman peak broadening.

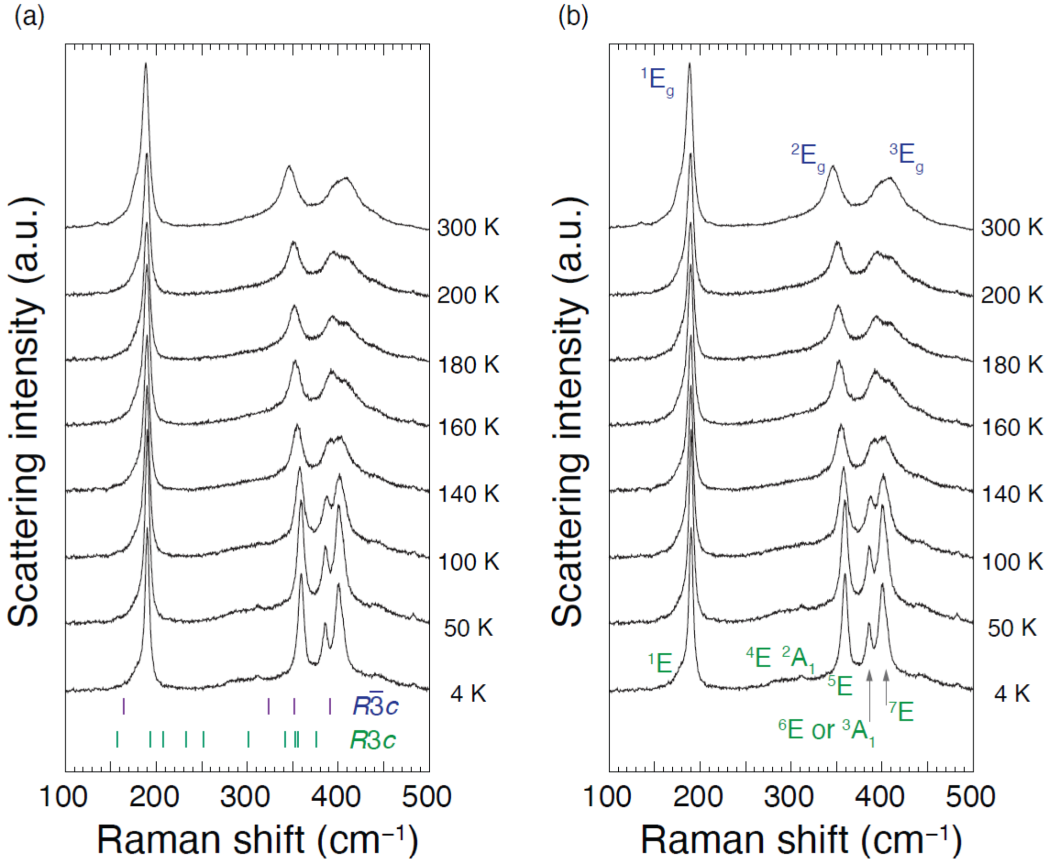


**Fig. S5**. Experimental Raman spectra of $LiReO_3$: (a) Calculated Raman shift positions for the $R\bar{3}c$ and $R3c$ structures, indicated by vertical bars below the spectra. (b) Raman peaks labeled according to their mode symmetry representations.

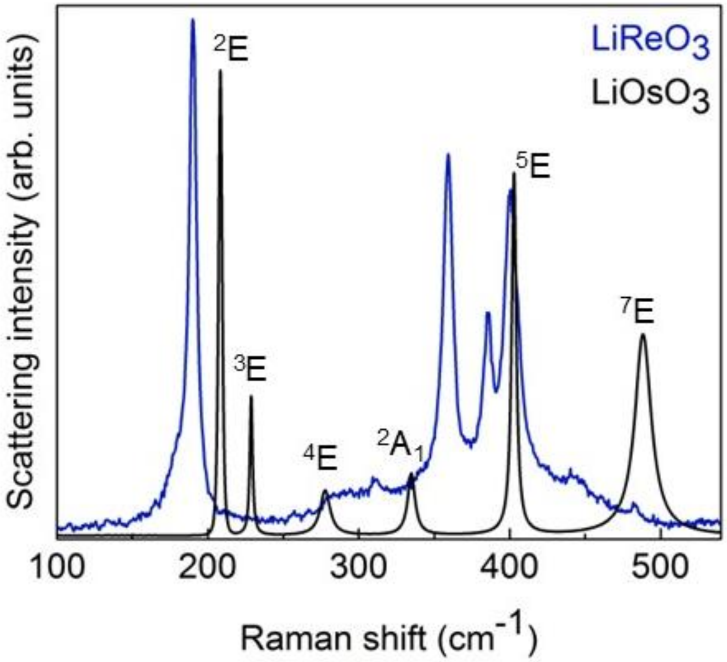

**Fig. S6**. Raman spectra of $LiReO_3$ (4 K) and $LiOsO_3$ (10 K), obtained below their P-NP phase transition temperatures. The data of $LiOsO_3$ is taken from Ref. 50. Labels denote the mode symmetry representations of the principal Raman peaks observed in $LiOsO_3$. $LiReO_3$ exhibits broader peaks and diffuse spectral intensity across a wider frequency range compared to $LiOsO_3$.

**Table S4**. Experimental and calculated Raman shifts for $LiOsO_3$ and $LiReO_3$ at 300 K, above their P-NP phase transition temperatures $T_s$. Above $T_s$, both compounds crystallize in the $R\overline{3}c$ structure with five Raman-active modes (one $A_{1g}$ and four $E_g$ modes). The experimental data for $LiOsO_3$ were taken from Ref. 48. Our calculated Raman-active mode frequencies for $LiOsO_3$, shown in this table, are in good agreement with previous calculations (*48*). The minor discrepancies, within 10 – 30 $cm^{-1}$ (0.0012 – 0.0037 eV), likely arise from differences in computational setting, such as pseudopotential type, exchange-correlation functional, and convergence criteria. Note that the Raman shift corresponding to the $^1A_{1g}$ mode was not assigned to be observed in $LiOsO_3$ (*48 – 50*). Raman data of $LiReO_3$ were taken in the range between 25 $cm^{-1}$ and 525 $cm^{-1}$ (see the main text of this paper).

| | $LiOsO_3$ | | $LiReO_3$ | |
|---|---|---|---|---|
| Mode | Calc. | Exp. | Calc. | Exp. |
| $^1A_{1g}$ | 423 | | 352 | |
| $^1E_g$ | 189 | 206 | 165 | 189 |
| $^2E_g$ | 380 | 402 | 323 | 344 |
| $^3E_g$ | 529 | 492 | 391 | 403 |
| $^4E_g$ | 633 | 643 | 659 | |

**Table S5**. Comparison of experimental Raman shifts (25 ~ 525 $cm^{-1}$) below the phase transition temperature with calculated values for the *R*3*c* structure. Thirteen Raman-active modes (four $A_1$ and nine *E* modes) are present in the *R*3*c* structure of $LiReO_3$. The calculated Raman-active mode frequencies agree well with experimental values, with precision comparable to that achieved for prior Raman studies of $LiNbO_3$ and $LiOsO_3$ (*24*, *48*).

| Mode | Cal. | Exp. |
|---|---|---|
| $^1A_1$ | 208 | |
| $^2A_1$ | 302 | 312 |
| $^3A_1$ | 355 | 387 |
| $^4A_1$ | 635 | |
| $^1E$ | 157 | 190 |
| $^2E$ | 193 | |
| $^3E$ | 233 | |
| $^4E$ | 252 | 281 |
| $^5E$ | 341 | 360 |
| $^6E$ | 353 | 387 |
| $^7E$ | 376 | 401 |
| $^8E$ | 658 | |
| $^9E$ | 667 | |

SXRD patterns of $LiRe_{1-x}Nb_xO_3$ samples

Here we present detailed results of SXRD patterns for niobium-substituted $LiRe_{1-x}Nb_xO_3$ samples. The variations and temperature profiles of the lattice parameters *a* and *c* are presented in Fig. 2A and 2B of the main text.

Temperature-dependent SXRD of $LiRe_{1-x}Nb_xO_3$ was measured at 100 K (left). Peaks of 012 (center) and 006 (right) were zoomed to trace the *a*- and *c*-axes along Nb-substitution. The slight impurities of $ReO_2$ and unknown rhombohedral impurity observed in $x = 0$ ($LiReO_3$) disappeared in all Nb-substituted samples. Zoomed plots are to watch the Nb-substitution behaviors of the 012 and 006 peaks that respectively correspond to *a*- and *c*-axis evolutions of the rhombohedral cell. Both peaks gradually shift from $x = 0$ ($LiReO_3$) to $x = 1$ ($LiNbO_3$), showing that the crystal lattice expanded in almost isotropic trend by Nb-substitution for Re due to the larger Nb in ionic radius (*79*).

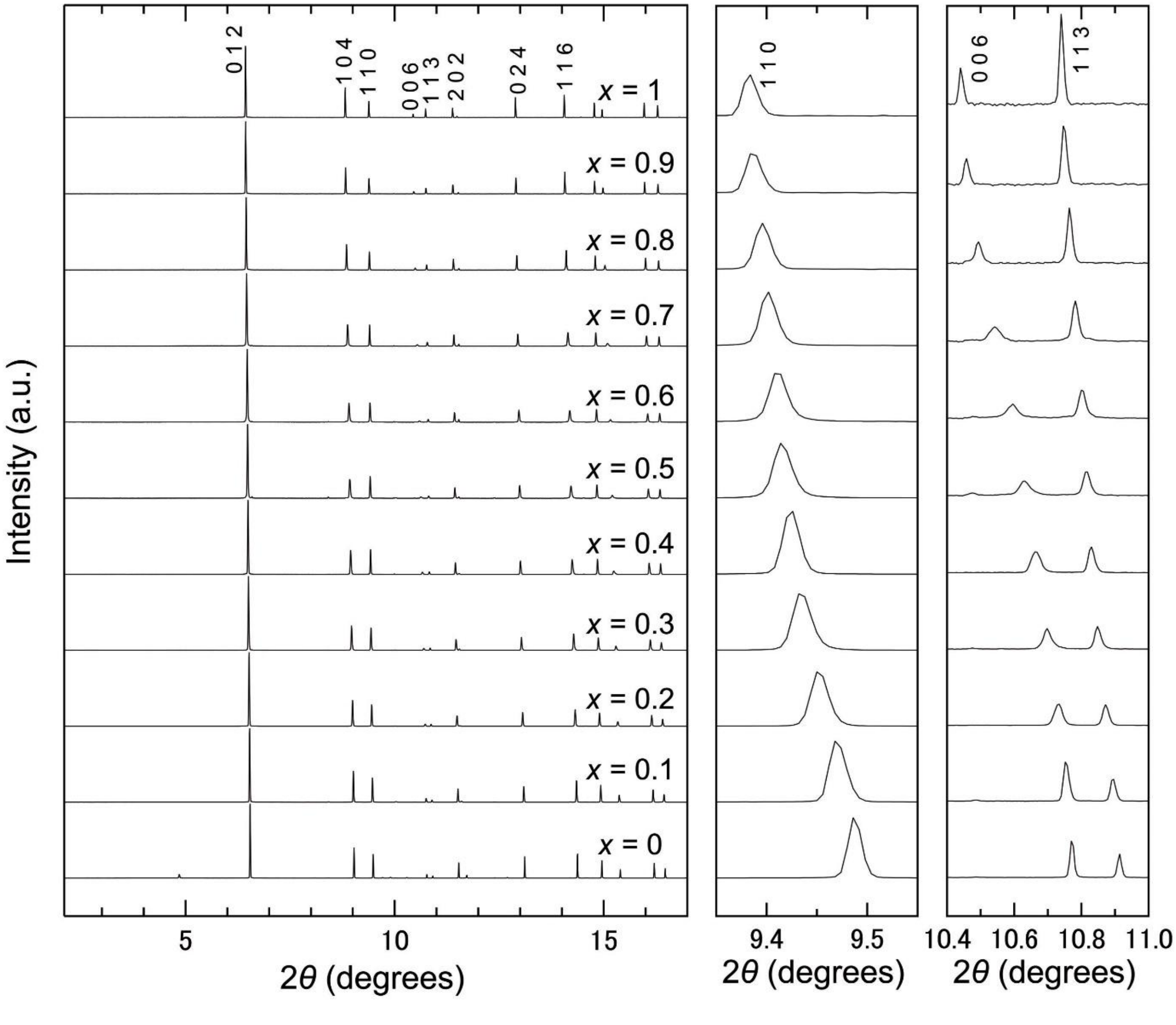


**Fig. S7.** SXRD patterns of $LiRe_{1-x}Nb_xO_3$ collected at 100 K. The overview of the patterns from 2 to 17 degrees (left). 012 (center) and 006 (right) peaks respectively point out the *ab*-plane and *c*-axis axes of the hexagonal rhombohedral unit cell, respectively.

Lattice evolutions of $LiRe_{1-x}Nb_xO_3$ samples ($x$ = 0.1, 0.2)

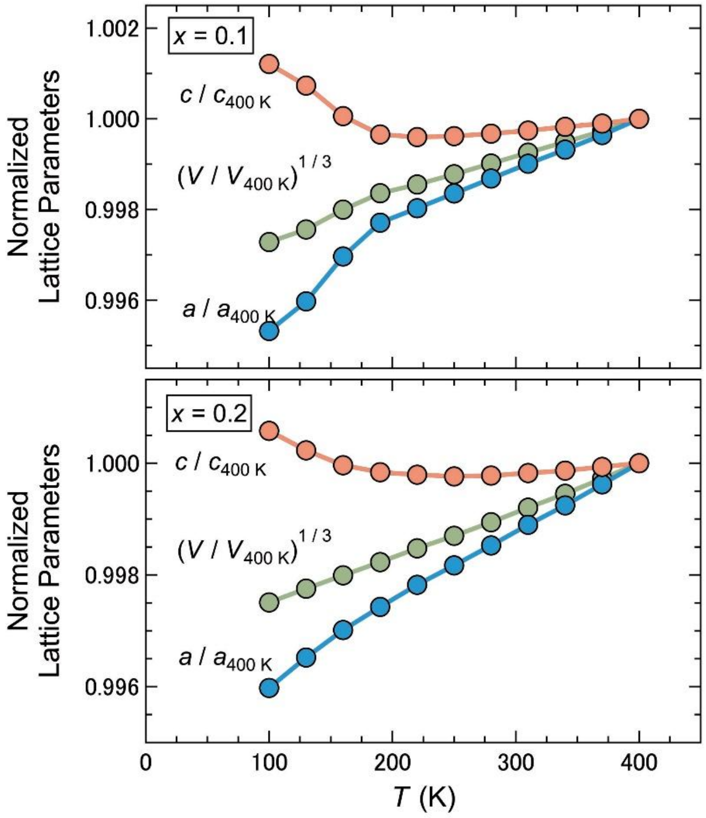


**Fig. S8.** Temperature-dependence of lattice parameters normalized at 400 K to 100 K. The top and bottom are the data of samples $x$ = 0.1 and 0.2 ($LiRe_{1-x}Nb_xO_3$), respectively.

Specific heat measurements of $LiRe_{1-x}Nb_xO_3$ samples ($x$ = 0.1, 0.2)

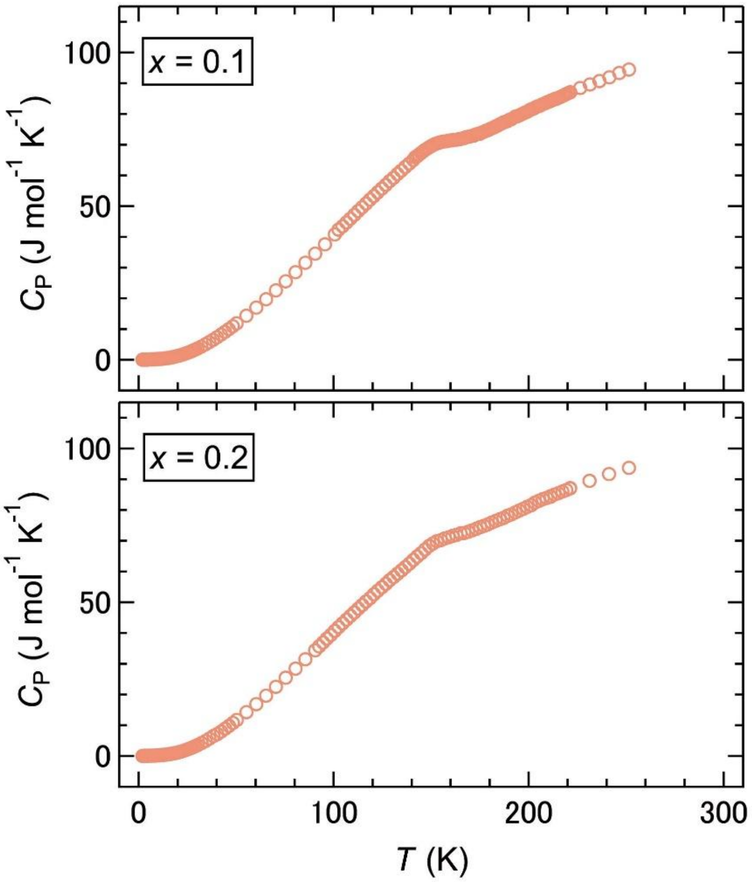


**Fig. S9.** Specific heats of $LiRe_{1-x}Nb_xO_3$ from 2 K to 250 K. The top and bottom are the data of samples $x$ = 0.1 and 0.2, respectively.

Thermal hysteresis of Raman scattering and resistivity experiments for $LiReO_3$

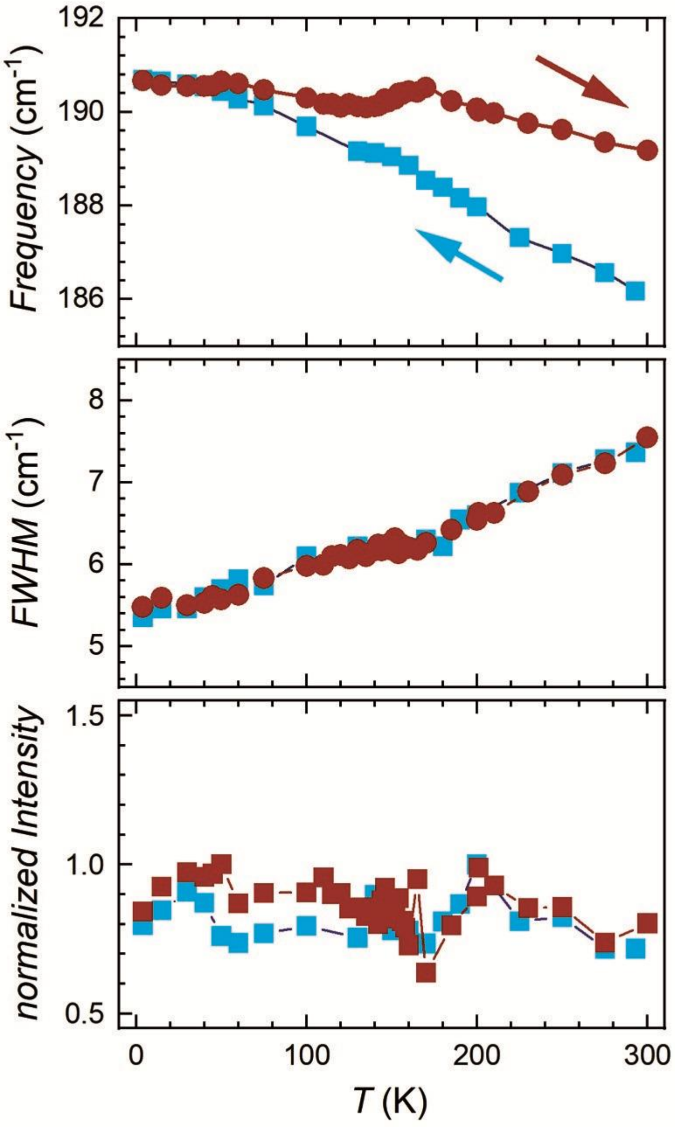


**Fig. S10.** Temperature-dependences of (top) frequency, (middle) full width at half maximum (FWHM) and (bottom) intensity of the Raman spectrum at the frequency of $^{1}E_{g}$ octahedral mode in $LiReO_3$. The frequency shows a deviation between the data collected on cooling and heating, indicating a part of the thermal hysteresis of the P-NP transition in metal. Other vibrational modes ($^{2}E_{g}$ and $^{3}E_{g}$) also exhibit similar thermal hysteresis with subtle variations in temperature range and loop width.

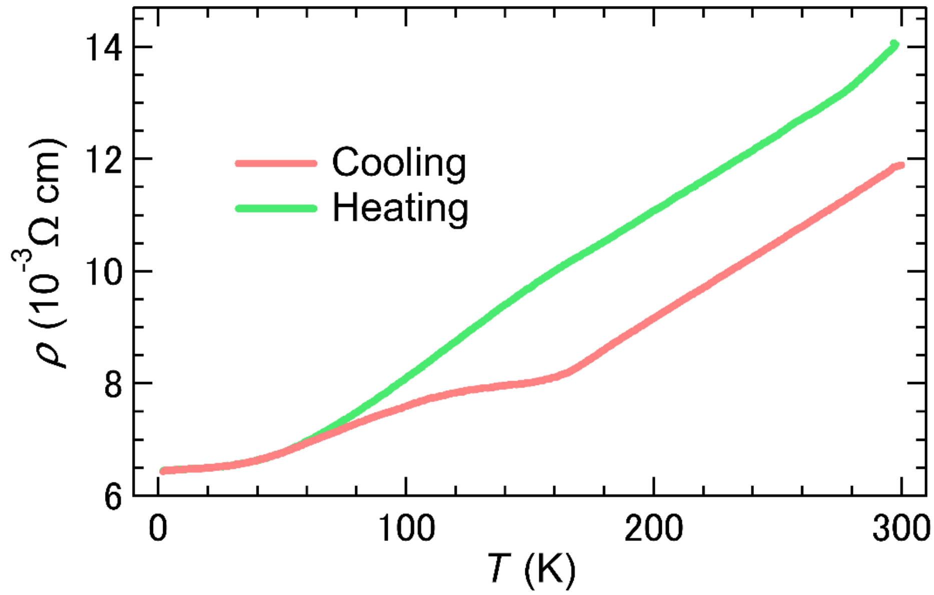


**Fig. S11.** Temperature dependence of electrical resistivity $\rho$ measured during cooling and heating cycles. The cooling and heating curves diverged at about 60 K. The hysteresis loop remained open to 300 K, as seen in the Raman shift frequency (fig. S10). Upon cooling, $\rho$ displays a kink at $T_s$ = 170 K, followed by a hump structure at lower temperatures, while these features remain subtle at elevated temperatures.

Thermal evolution of theoretical calculation for $LiNbO_3$

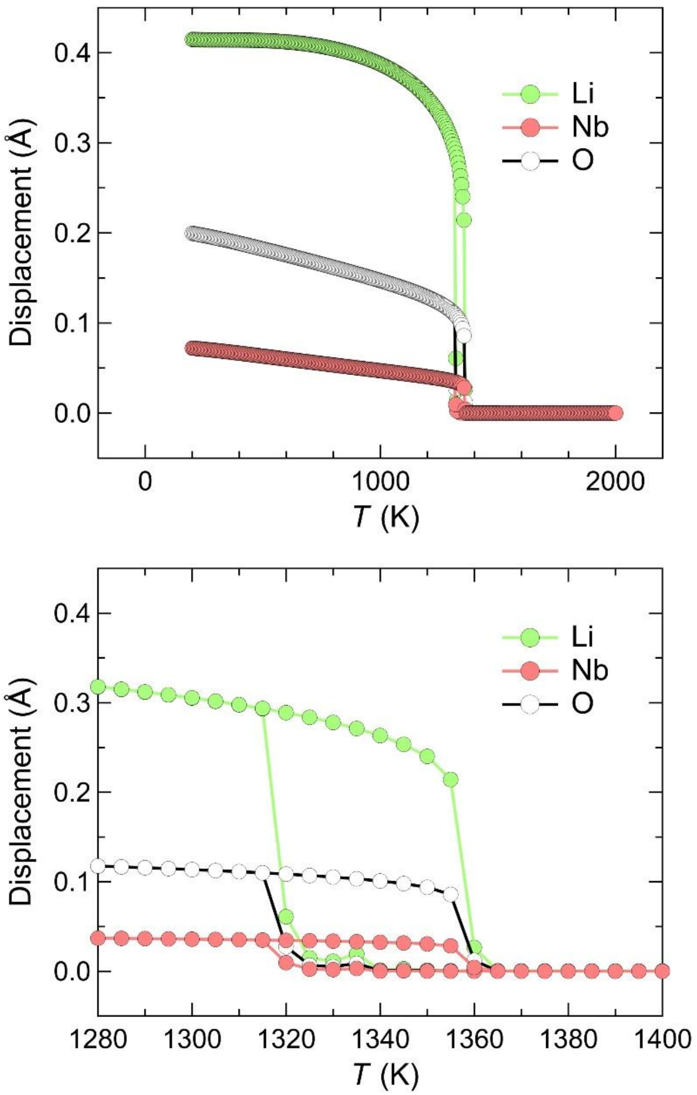


**Fig. S12.** Calculated atomic displacements of $LiNbO_3$ as a function of temperature (top). The green, red and white circles represent atomic displacements of Li, Nb and O, respectively. The heating and cooling treatments produced thermal hysteresis between 1320 K and 1360 K ($\Delta T_{calc}$ = 40 K) (bottom).

Entropy changes of $LiReO_3$ examined from specific heat measurements

The specific heat $C_P$ reveals a lambda-type peak at $T_s$ = 170 K (Fig. 1C of the main text), indicating a phase transition at macroscopic spatiotemporal scales in $LiReO_3$. In this section, we quantitatively evaluate the associated entropy change from the $C_P$ data. Specifically, the $C_P$ data shown in the middle of Fig. 1C was first fitted using a cubic polynomial function in the temperature range between 55 K and 230 K. This fitted curve ($C_{fit}$) was then subtracted from the measured $C_P$ in the range from 100 K to 200 K to estimate the entropy change during a phase transition.

The deviation $C_P - C_{fit}$ (green circles shown in fig. S13) has a peak at $T_s$ = 170 K and the transition entropy $\Delta S$ is 0.33$R$ (J $K^{-1}$) (approximately half of $R$ln2), calculated by integrating ($C_P - C_{fit}$)/$T$ from $T$ = 100 K to 200 K. The entropy loss measured here provides additional evidence for spatiotemporal fluctuations identified through ultrasonic and Raman scattering experiments (Fig. 5A and fig. S5).

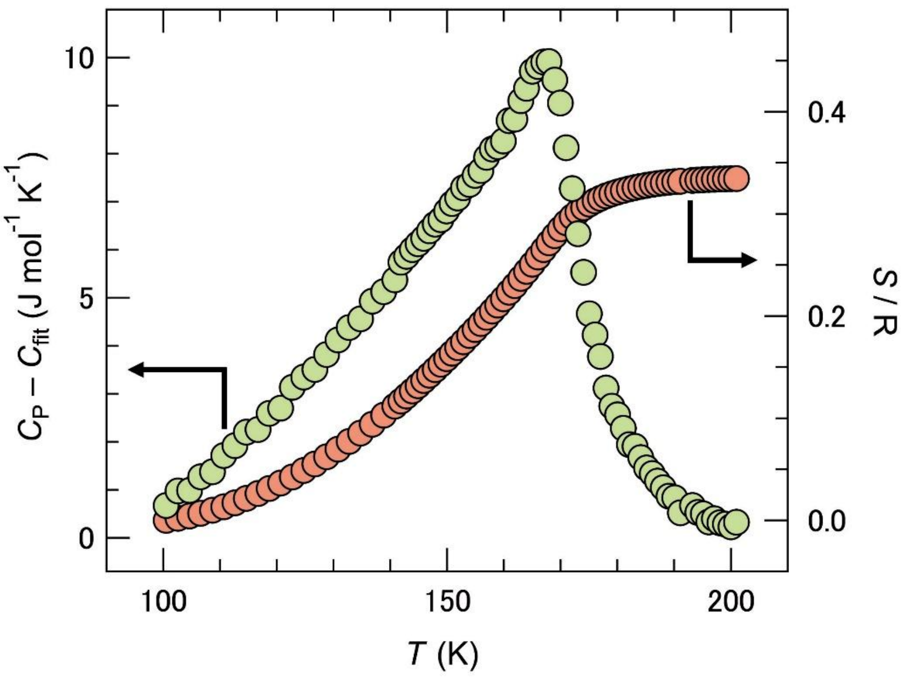


**Fig. S13.** The deviation of specific heat $C_P$ of $LiReO_3$ from the fit curve $C_{fit}$ (green circle) and the entropy change $S$ from 100 K divided by the universal gas constant $R$ (red circle).

The magnetic susceptibility measurements for $LiReO_3$

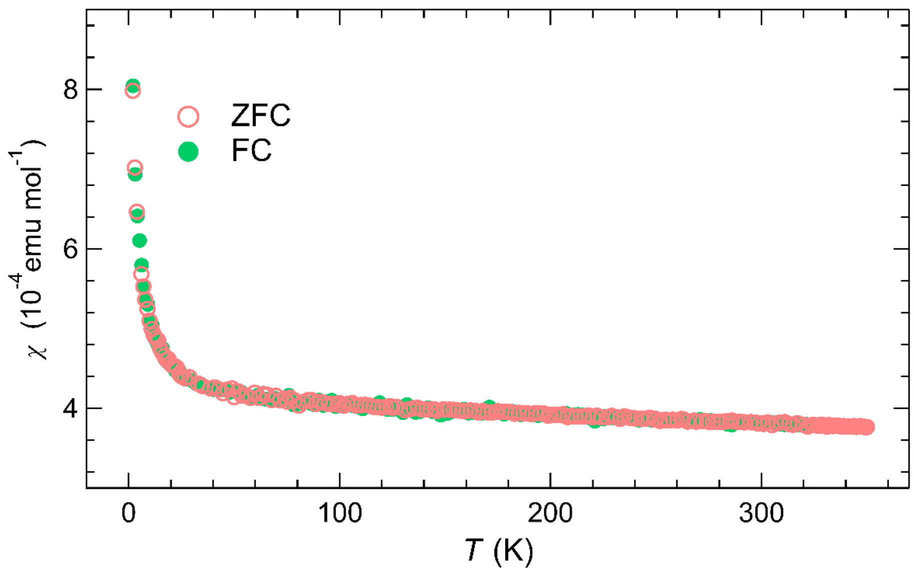


**Fig. S14.** Magnetic susceptibility of $LiReO_3$ at temperatures between 2 K and 350 K. No signature of magnetic ordering was observed in this temperature range.

Projected density of states (PDOS) of polar metallic $LiReO_3$

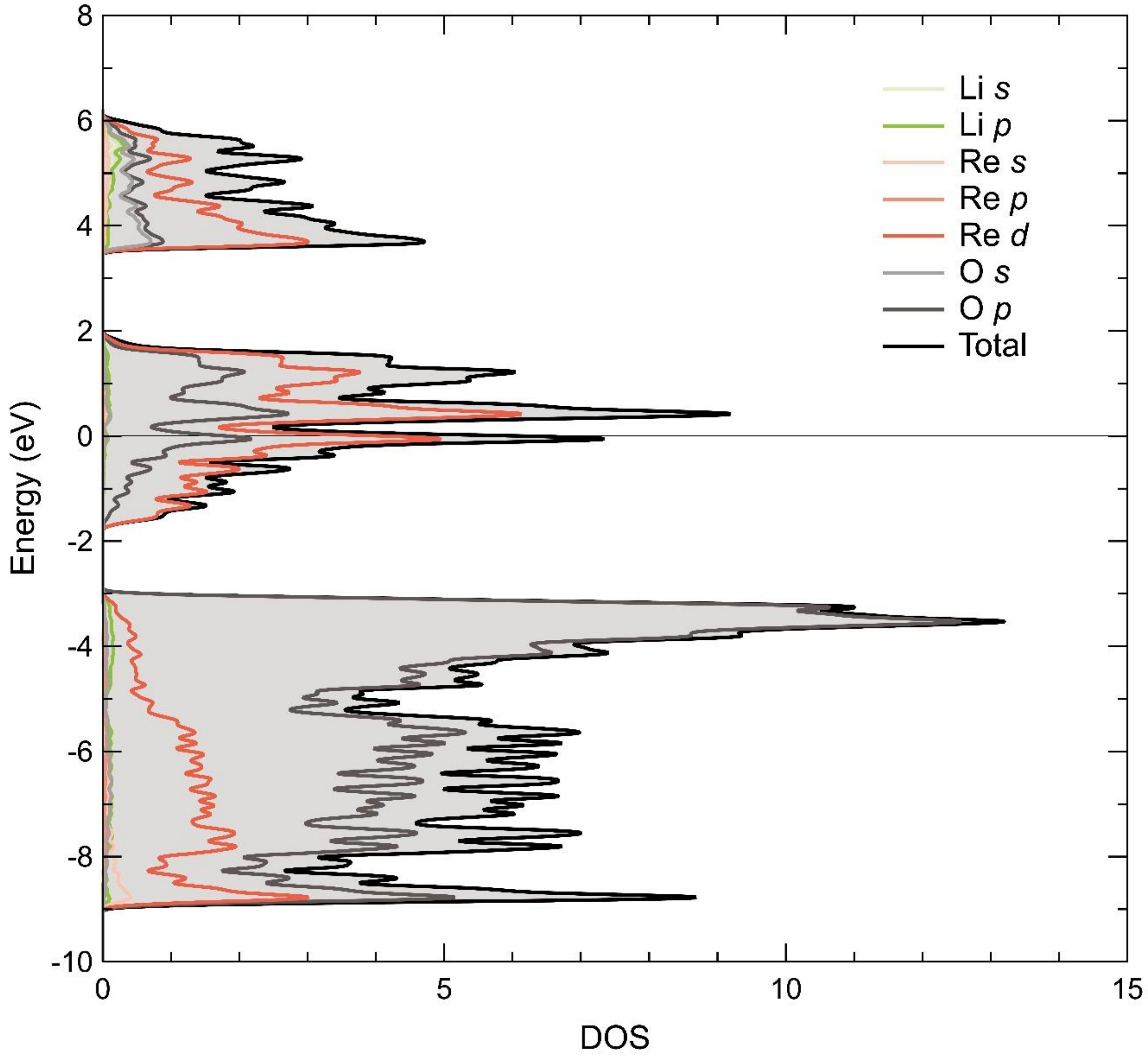


**Fig. S15.** PDOS of polar $LiReO_3$. The Fermi level (0 eV) is occupied by the Re 5*d* and O 2*p* orbitals, contributing to the metallic electronic state.

Temporal and spatial scales of fluctuations in $LiReO_3$

Probe-dependent measurements of hysteresis ranges (Fig. 3, figs. S10, S11), anomalous ultrasonic absorption-resonance behavior (Fig. 5A), and broad features of the Raman spectrum (fig. S6) indicate diffusive dynamics associated with spatiotemporal phase fluctuations in $LiReO_3$. In this section, we examine the temporal and spatial scales of these fluctuations based on experimental results for $LiReO_3$.

Spatial Fluctuations: Our study indicates that spatial fluctuations in $LiReO_3$ occur across a broad range of length scales. X-ray diffraction (XRD) measurements, which are sensitive to macroscopic separations, show no evidence of long-scale phase separation between the polar and nonpolar phases, suggesting that these fluctuations take place on relatively small scales. Considering the rhombohedral unit cell dimensions ($a$ = 5.1 Å, $c$ = 13.4 Å), the lower bound for spatial fluctuations would be several unit cells, i.e., 10 – 30 Å, representing the minimum size for coherent polar or nonpolar regions. On the upper end, ultrasonic experiments, assuming a sound velocity of 10,000 m/s and measurement frequencies of 20 ~ 100 MHz, suggest scattering centers on the order of $\lambda = v/f = 1 \sim 5 \times 10^{-4}$ m. These estimates indicate that spatial fluctuations span at least five orders of magnitude from tens of angstroms to hundreds of micrometers, depending on the experimental technique used.

Temporal Fluctuations: Temporal fluctuations also span multiple time scales. At longer time scales, low-temperature ultrasound experiments reveal resonant and absorptive behavior in the range of $0.1 \sim 10 \times 10^{-5}$ seconds (Fig. 5A), corresponding to 1 ~ 100 μs fluctuations. At shorter time scales, Raman scattering captures ion vibration dynamics between $10^{-12}$ and $10^{-13}$ s. Thus, temporal fluctuations span a wide range, from $1 \times 10^{-12}$ to $1 \times 10^{-6}$ s.

Further investigations into their spatial distribution in $LiReO_3$ remain an important future task. Experimentally capturing temporal fluctuations is challenging yet understanding how these fluctuations influence the functionality of polar metals is essential.

Supplemental insights into the phase transition of $LiReO_3$

We observe that $LiReO_3$ exhibits a first-order phase transition while displaying characteristic fluctuations across multiple temporal and spatial scales (Figs. 1C, 3A, 3B, 5A, figs. S6, S10, S11). In this section, we provide supplementary discussion of this diffusive phase transition phenomenon in $LiReO_3$, comparing it with previous cases of first-order phase transitions and well-known insulating relaxor ferroelectrics exhibiting phase separations and fluctuations. We also offer additional explanations concerning the behavior of the precursor temperature region in $LiReO_3$.

Previous studies of first-order transitions in solid-state materials, including systems exhibiting metal-insulator transitions and charge ordering, have focused on the spatial distribution of coexisting phases (*83–88*, *115*, *116*). In these systems, precursor phenomena and diffuse dynamics above transition temperatures have been investigated extensively in terms of phase separations. Diffusive behavior and phase transitions have also been studied in insulating polar materials such as relaxor ferroelectrics (*7*). These transitions of relaxors are characterized by a broad peak in dielectric permittivity across a wide temperature range associated with the continuous development of polar nanoregions (PNRs), as seen in $PbMg_{1/3}Nb_{2/3}O_3$ (*117*) and $PbSc_{0.5}Ta_{0.5}O_3$ (*118*). $LiReO_3$, however, exhibits a fundamentally different mechanism where its diffusive transition arises from competing phases rather than chemical disorder as observed in manganese oxides (*119*) and relaxors (*7*, *117*, *118*). In $LiReO_3$, the polar and nonpolar structural phases dynamically fluctuate, coexist, and compete across diverse temporal and spatial scales. This phase fluctuation is facilitated by a shallow energy potential (Fig. 4), influenced by conduction electrons, suggesting their important role in the phase transition and fluctuation of this polar metallic system.

A precursor phenomenon of phase separations may be observed in the transverse elastic constant of $LiReO_3$ (Fig. 3B), where hardening behavior is observed above $T_s$ = 170 K. The increase in elastic constant of $LiReO_3$ (about 20%) toward $T_s$ is 5 – 10 times larger than observed in conventional materials (*61*, *100*, *101*), suggesting additional effects beyond the suppression of phonon anharmonicity during cooling. While materials with second-order phase transitions typically exhibit elastic softening toward transition temperatures (*60*), hardening has been reported in materials with first-order phase transitions, where embryonic domain formation plays a role (*102–104*). These precursor-related effects likely contribute to the hardening observed above $T_s$ in $LiReO_3$, consistent with the first-order nature of its phase transition (Figs. 3, 4, fig. S16).

Details of theoretical calculations

In this section, we outline the parameters and conditions of our theoretical calculations for understanding the phase transition behavior of $LiReO_3$, including the phonon calculations used in Raman spectra analysis.

The 2 × 2 × 2 supercell, which contains 80 atoms, is used for phonon calculations of $LiReO_3$ and $LiNbO_3$. The used lattice constants are $a$ = 5.126656 Å and $c$ = 13.369952 Å for $LiReO_3$, and $a$ = 5.18178 Å and $c$ = 13.631355 Å for $LiNbO_3$, which were obtained by structural optimization implemented in Vienna Ab initio Simulation Package (VASP) (*99*).

The harmonic interatomic force constants (IFCs) are calculated using the finite-displacement method with the displacement magnitude of 0.01 Angstrom. The anharmonic IFCs, which are truncated in the fourth order, are calculated using the compressive sensing method (*42*, *120*) from the displacement-force data. To obtain the configurations of the displacement force data, we use *ab* initio molecular dynamics (AIMD) to sample the low-energy region of the potential energy surface.

The Γ-point phonon frequencies and eigenvectors were calculated for $LiReO_3$ from the harmonic IFCs with PHONOPY code (*121*, *122*), and the irreducible representations of the eigenmodes were identified. Raman active phonon modes were selected according to the irreducible representations to be compared with the experimental Raman spectra (fig. S5). For $LiReO_3$, AIMD is performed at 300 K for 16000 fs with the step of 1.0 fs. The first 1000 steps are discarded as thermalization steps, and 300 snapshots are extracted with the interval of 50 steps from the remaining 15000 steps. We generate the configurations by adding random atomic displacements of 0.04 Å to these snapshots. Similarly, we run the AIMD at 500 K and 750 K for $LiNbO_3$. The calculations are performed for 8000 fs with the step of 1 fs. At each temperature, we discard the first 1000 steps as the thermalization steps and generate 140 configurations, like in $LiReO_3$.

The temperatures of the AIMD calculations are controlled by velocity scaling. The cutoff energy of 400 eV and 2 × 2 × 2 Monkhorst-Pack $k$-mesh are used for the AIMD calculations. We use the small cutoff energy and the coarse $k$-mesh because the AIMD results are used only for generating relevant atomic configurations at finite temperatures.

We perform DFT calculations of the obtained configurations to generate the displacement-force data. The cutoff energy of 600 eV and 2 × 2 × 2 Monkhorst-Pack $k$-mesh is employed at this stage to get accurate results. The AIMD calculations and the DFT calculations are performed using VASP (*99*). We use PBEsol exchange-correlation functional and PAW pseudopotentials (*94–96*, *123*).

The anharmonic IFCs are calculated from the obtained displacement-force data using the compressive sensing method implemented in the ALAMODE package (*42*, *97*, *98*). Here, we set the cutoff radius of the cubic and quartic IFCs were set at 12.0 bohr and 10.0 bohr, respectively. The quartic IFCs are limited to up to three-body terms to improve computational efficiency. The fitting errors are 6.97% for $LiReO_3$ and 12.62% for $LiNbO_3$. We use 8 × 8 × 8 $q$-mesh for the SCP calculation.

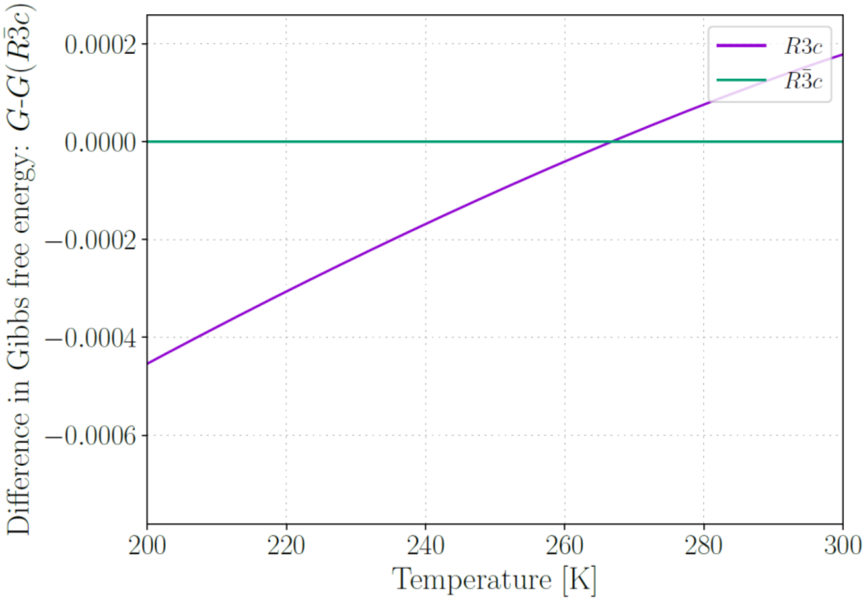


**Fig. S16.** Temperature dependence of Gibbs free energy in $LiReO_3$. The crossing of Gibbs energy curves at the transition temperature characterizes the first-order nature of the phase transformation.

## REFERENCES


1. P. S. Halasyamani, K. R. Poeppelmeier, Noncentrosymmetric oxides. *Chem. Mater.* **10**, 2753–2769 (1998).

2. R. S. Weis, T. K. Gaylord, Lithium niobate: Summary of physical properties and crystal structure. *Appl. Phys. A* **37**, 191–203 (1985).

3. B. Naranjo, J. K. Gimzewski, S. Putterman, Observation of nuclear fusion driven by a pyroelectric crystal. *Nature* **434**, 1115–1117 (2005).

4. H. Boysen, F. Altorfer, A neutron powder investigation of the high-temperature structure and phase transition in $LiNbO_3$. *Acta Crystallogr. Sect. B* **50**, 405–414 (1994).

5. M. R. Chowdhurytt, G. E. Peckhamt, D. H. Saunderson, A neutron inelastic scattering study of $LiNbO_3$. *J. Phys. C Solid State Phys.* **11**, 1671–1683 (1978).

6. Y. Kuno, C. Tassel, K. Fujita, D. Batuk, A. M. Abakumov, K. Shitara, A. Kuwabara, H. Moriwake, D. Watabe, C. Ritter, C. M. Brown, T. Yamamoto, F. Takeiri, R. Abe, Y. Kobayashi, K. Tanaka, H. Kageyama, $ZnTaO_2N$: Stabilized high-temperature $LiNbO_3$-type structure. *J. Am. Chem. Soc.* **138**, 15950–15955 (2016).

7. L. Eric Cross, Relaxor ferroelectirics. *Ferroelectrics* **76**, 241–267 (1987).

8. M. E. Manley, J. W. Lynn, D. L. Abernathy, E. D. Specht, O. Delaire, A. R. Bishop, R. Sahul, J. D. Budai, Phonon localization drives polar nanoregions in a relaxor ferroelectric. *Nat. Commun.* **5**, 3683 (2014).

9. A. Aimi, T. Katsumata, D. Mori, D. Fu, M. Itoh, T. Kyômen, K. I. Hiraki, T. Takahashi, Y. Inaguma, High-pressure synthesis and correlation between structure, magnetic, and dielectric properties in $LiNbO_3$-type $MnMO_3$ (M = Ti, Sn). *Inorg. Chem.* **50**, 6392–6398 (2011).

10. T. Varga, A. Kumar, E. Vlahos, S. Denev, M. Park, S. Hong, T. Sanehira, Y. Wang, C. J. Fennie, S. K. Streiffer, X. Ke, P. Schiffer, V. Gopalan, J. F. Mitchell, Coexistence of weak

ferromagnetism and ferroelectricity in the high pressure $LiNbO_3$-type phase of $FeTiO_3$. *Phys. Rev. Lett.* **103**, 047601 (2009).

11. P. W. Anderson, E. I. Blount, Symmetry considerations on martensitic transformations: "Ferroelectric" metals? *Phys. Rev. Lett.* **14**, 217–219 (1965).

12. Y. Shi, Y. Guo, X. Wang, A. J. Princep, D. Khalyavin, P. Manuel, Y. Michiue, A. Sato, K. Tsuda, S. Yu, M. Arai, Y. Shirako, M. Akaogi, N. Wang, K. Yamaura, A. T. Boothroyd, Ferroelectric-like structural transition in a metal. *Nat. Mater.* **12**, 1024–1027 (2013).

13. H. J. Xiang, Origin of polar distortion in $LiNbO_3$-type "ferroelectric" metals: Role of A-site instability and short-range interactions. *Phys. Rev. B* **90**, 094108 (2014).

14. G. Giovannetti, M. Capone, Dual nature of the ferroelectric and metallic state in $LiOsO_3$. *Phys. Rev. B* **90**, 195113 (2014).

15. W. X. Zhou, A. Ariando, Review on ferroelectric/polar metals. *Jpn. J. Appl. Phys.* **59**, SI0802 (2020).

16. D. Hickox-Young, D. Puggioni, J. M. Rondinelli, Polar metals taxonomy for materials classification and discovery. *Phys. Rev. Mater.* **7**, 010301 (2023).

17. N. J. Laurita, A. Ron, J. Y. Shan, D. Puggioni, N. Z. Koocher, K. Yamaura, Y. Shi, J. M. Rondinelli, D. Hsieh, Evidence for the weakly coupled electron mechanism in an Anderson-Blount polar metal. *Nat. Commun.* **10**, 3217 (2019).

18. J. J. Gao, S. Y. Fu, K. Yamaura, J. F. Lin, J.-S. Zhou, Room-temperature polar metal stabilized under high pressure. *Phys. Rev. B* **101**, 220101(R) (2020).

19. J. Y. Shan, A. de la Torre, N. J. Laurita, L. Zhao, C. D. Dashwood, D. Puggioni, C. X. Wang, K. Yamaura, Y. Shi, J. M. Rondinelli, D. Hsieh, Evidence for an extended critical fluctuation region above the polar ordering transition in $LiOsO_3$. *Phys. Rev. Res.* **2**, 033174 (2020).

20. A. Jayaraman, A. A. Ballman, Effect of pressure on the Raman modes in $LiNbO_3$ and $LiTaO_3$. *J. Appl. Phys.* **60**, 1208–1210 (1986).

21. I. Inbar, R. E. Cohen, Comparison of the electronic structures and energetics of ferroelectric $LiNbO_3$ and $LiTaO_3$. *Phys. Rev. B* **53**, 1193–1204 (1996).

22. A. Ridah, M. D. Fontana, P. Bourson, Temperature dependence of the Raman modes in $LiNbO_3$ and mechanism of the phase transition. *Phys. Rev. B* **56**, 5967–5973 (1997).

23. F. P. Safaryan, On the theory of ferroelectric transition in the crystal $LiNbO_3$. *Phys. Lett. A* **255**, 191–200 (1999).

24. K. Parlinski, Z. Q. Li, Y. Kawazoe, Ab initio calculations of phonons in $LiNbO_3$. *Phys. Rev. B* **61**, 272–278 (2000).

25. J.-S. Zhou, X. Li, J. M. He, J. Ch, K. Yamaura, Strongly correlated electrons in the ferroelectric metal $LiOsO_3$. *Phys. Rev. B* **104**, 115130 (2021).

26. R. J. Cava, A. Santoro, D. W. Murphy, S. Zahurak, R. S. Roth, The structures of lithium-inserted metal oxides: $LiReO_3$ and $Li_2ReO_3$. *J. Solid State Chem.* **42**, 251–262 (1982).

27. Y. Ikeuchi, H. Takatsu, C. Tassel, Y. Goto, T. Murakami, H. Kageyama, High-pressure synthesis of fully occupied tetragonal and cubic tungsten bronze oxides. *Angew. Chem. Int. Ed. Engl.* **129**, 5864–5867 (2017).

28. Y. Ikeuchi, H. Takatsu, C. Tassel, C. M. Brown, T. Murakami, Y. Matsumoto, Y. Okamoto, H. Kageyama, Rattling behavior in a simple perovskite $NaWO_3$. *Inorg. Chem.* **58**, 6790–6795 (2019).

29. K. Ishida, Y. Ikeuchi, C. Tassel, H. Takatsu, C. M. Brown, H. Kageyama, High-pressure synthesis of non-stoichiometric $Li_xWO_3$ ($0.5 \leq x \leq 1.0$) with $LiNbO_3$ structure. *Inorganics* **7**, 10.3390/inorganics7050063 (2019).

30. S. C. Abrahams, J. M. Reddy, J. L. Bernstein, Ferroelectric lithium niobate. 3. Single crystal x-ray diffraction study at 24°C. *J. Phys. Chem. Solids* **27**, 997–1012 (1966).

31. S. C. Abrahams, H. J. Levinstein, J. M. Reddy, Ferroelectric lithium niobate. 5. Polycrystal x-ray diffraction study between 24°C and 1200°C. *J. Phys. Chem. Solids* **27**, 1019–1026 (1966).

32. S. C. Abrahams, J. L. Bernstein, Ferroelectric lithium tantalate−1. Single crystal x-ray diffraction study at 24 °C. *J. Phys. Chem. Solids* **28**, 1685–1692 (1967).

33. S. C. Abrahams, E. Buehler, W. C. Hamilton, S. J. Laplaca, Ferroelectric lithium tantalate-III. Temperature depednece of the structure in the ferroelectric phase and the paraelectric structure at 940°K. *J. Phys. Chem. Solids* **34**, 521–532 (1973).

34. M. Cěh, D. Kolar, L. Golič, The phase diagram of $CaTiO_3$-$SrTiO_3$. *J. Solid State Chem.* **68**, 68–72 (1987).

35. M. McQuarrie, Structural behavior in the system (Ba, Ca, Sr)$TiO_3$, and its relation to certain dielectric characteristics. *J. Am. Ceram. Soc.* **38**, 444–449 (1955).

36. G. Durst, M. Grotenhuis, A. G. Barkow, Solid solubility study of barium, strontium, and calcium titanates. *J. Am. Ceram. Soc.* **33**, 133–139 (1950).

37. J. A. Basmajian, R. C. DeVries, Phase equilibria in the system $BaTiO_3$–$SrTiO_3$. *J. Am. Ceram. Soc.* **40**, 373–376 (1957).

38. H. Sakai, K. Ikeura, M. S. Bahramy, N. Ogawa, D. Hashizume, J. Fujioka, Y. Tokura, S. Ishiwata, Critical enhancement of thermopower in a chemically tuned polar semimetal $MoTe_2$. *Sci. Adv.* **2**, e1601378 (2016).

39. T. Goto, Y. Nemoto, K. Sakai, T. Yamaguchi, M. Akatsu, T. Yanagisawa, H. Hazama, K. Onuki, H. Sugawara, H. Sato, Quadrupolar effect and rattling motion in the heavy-fermion superconductor $PrOs_4Sb_{12}$. *Phys. Rev. B* **69**, 180511(R) (2004).

40. Y. Nambu, J. S. Gardner, D. E. MacLaughlin, C. Stock, H. Endo, S. Jonas, T. J. Sato, S. Nakatsuji, C. Broholm, Spin fluctuations from hertz to terahertz on a triangular lattice. *Phys. Rev. Lett.* **115**, 127202 (2015).

41. D. J. Hooton, The use of a model in anharmonic lattice dynamics. *Philos. Mag.* **3**, 49–54 (1958).

42. T. Tadano, S. Tsuneyuki, Self-consistent phonon calculations of lattice dynamical properties in cubic $SrTiO_3$ with first-principles anharmonic force constants. *Phys. Rev. B* **92**, 054301 (2015).

43. T. Tadano, S. Tsuneyuki, First-principles lattice dynamics method for strongly anharmonic crystals. *J. Phys. Soc. Jpn.* **87**, 041015 (2018).

44. L. Monacelli, R. Bianco, M. Cherubini, M. Calandra, I. Errea, F. Mauri, The stochastic self-consistent harmonic approximation: Calculating vibrational properties of materials with full quantum and anharmonic effects. *J. Phys. Condens. Matter* **33**, 363001 (2021).

45. A. van Roekeghem, J. Carrete, N. Mingo, Quantum self-consistent ab-initio lattice dynamics. *Comput. Phys. Commun.* **263**, 107945 (2021).

46. R. Masuki, T. Nomoto, R. Arita, T. Tadano, Ab-initio structural optimization at finite temperatures based on anharmonic phonon theory: Application to the structural phase transitions of $BaTiO_3$. *Phys. Rev. B* **106**, 224104 (2022).

47. R. Masuki, T. Nomoto, R. Arita, T. Tadano, Full optimization of quasiharmonic free energy with an anharmonic lattice model: Application to thermal expansion and pyroelectricity of wurtzite GaN and ZnO. *Phys. Rev. B* **107**, 134119 (2023).

48. F. Jin, A. Zhang, J. Ji, K. Liu, L. Wang, Y. Shi, Y. Tian, X. Ma, Q. Zhang, Raman phonons in the ferroelectric-like metal $LiOsO_3$. *Phys. Rev. B* **93**, 064303 (2016).

49. H. Padmanabhan, Y. Park, D. Puggioni, Y. Yuan, Y. Cao, L. Gasparov, Y. Shi, J. Chakhalian, J. M. Rondinelli, V. Gopalan, Linear and nonlinear optical probe of the ferroelectric-like phase transition in a polar metal, $LiOsO_3$. *Appl. Phys. Lett.* **113**, 122906 (2018).

50. F. Jin, L. Wang, A. Zhang, J. Ji, Y. Shi, X. Wang, R. Yu, J. Zhang, E. W. Plummer, Q. Zhang, Raman interrogation of the ferroelectric phase transition in polar metal $LiOsO_3$. *Proc. Natl. Acad. Sci. U.S.A.* **116**, 20322–20327 (2019).

51. R. E. Cohen, H. Krakauer, Lattice dynamics and origin of ferroelectricity in $BaTiO_3$: Linearized-augmented-plane-wave total-energy calculations. *Phys. Rev. B* **42**, 6416–6423 (1990).

52. R. E. Cohen, Origin of ferroelectricity in perovskite oxides. *Nature* **358**, 136–138 (1992).

53. M. Acosta, N. Novak, V. Rojas, S. Patel, R. Vaish, J. Koruza, G. A. Rossetti Jr., J. Rodel, $BaTiO_3$-based piezoelectrics: Fundamentals, current status, and perspectives. *Appl. Phys. Rev.* **4**, 041305 (2017).

54. J. M. Ziman, *Electrons and Phonons* (Oxford Univ. Press, 1960).

55. T. Moriya, *Spin Fluctuations in Itinerant Electron Magnetism* (Springer-Verlag, 1985).

56. T. Kasuya, Effects of *s-d* interaction on transport phenomena. *Prog. Theor. Phys.* **22**, 227–246 (1959).

57. K. Otsuka, X. Ren, Physical metallurgy of Ti-Ni-based shape memory alloys. *Prog. Mater. Sci.* **50**, 511–678 (2005).

58. D. A. Keen, A. L. Goodwin, The crystallography of correlated disorder. *Nature* **521**, 303–309 (2015).

59. H. Kageyama, K. Hayashi, K. Maeda, J. P. Attfield, Z. Hiroi, J. M. Rondinelli, K. R. Poeppelmeier, Expanding frontiers in materials chemistry and physics with multiple anions. *Nat. Commun.* **9**, 772 (2018).

60. M. A. Carpenter, E. K. H. Salje, Elastic anomalies in minerals due to structural phase transitions. *Eur. J. Mineral.* **10**, 693–812 (1998).

61. B. Lüthi, *Physical Acoustics in the Solid State* (Springer Science & Business Media, 2005).

62. S. R. Phillpot, V. Gopalan, Coupled displacive and order-disorder dynamics in by molecular-dynamics simulation. *Appl. Phys. Lett.* **84**, 1916–1918 (2004).

63. S. Sanna, W. G. Schmidt, Ferroelectric phase transition in $LiNbO_3$: Insights from molecular dynamics. *IEEE Trans. Ultra. Ferroelectr. Freq. Control* **59**, 1925–1928 (2012).

64. S. Sanna, S. Neufeld, M. Rüsing, G. Berth, A. Zrenner, W. G. Schmidt, Raman scattering efficiency in $LiTaO_3$ and $LiNbO_3$ crystals. *Phys. Rev. B* **91**, 224302 (2015).

65. S. Margueron, A. Bartasyte, A. M. Glazer, E. Simon, J. Hlinka, I. Gregora, J. Gleize, Resolved E-symmetry zone-centre phonons in $LiTaO_3$ and $LiNbO_3$. *J. Appl. Phys.* **111**, 1041051 (2012).

66. E. Dagotto, Complexity in strongly correlated electronic systems. *Science* **309**, 257–262 (2005).

67. S. A. Kivelson, E. Fradkin, V. J. Emery, Electronic liquid-crystal phases of a doped Mott insulator. *Nature* **393**, 550–553 (1998).

68. B. Keimer, S. A. Kivelson, M. R. Norman, S. Uchida, J. Zaanen, From quantum matter to high-temperature superconductivity in copper oxides. *Nature* **518**, 179–186 (2015).

69. Y. Sato, S. Kasahara, H. Murayama, Y. Kasahara, E. Moon, T. Nishizaki, T. Loew, J. Porras, B. Keimer, T. Shibauchi, Y. Matsuda, Thermodynamic evidence for a nematic phase transition at the onset of the pseudogap in $YBa_2Cu_3O_y$. *Nat. Phys.* **13**, 1074–1078 (2017).

70. S. M. Shapiro, Y. Noda, Y. Fujii, Y. Yamada, X-ray investigation of the premartensitic phase in $Ni_{46.8}Ti_{50}Fe_{3.2}$. *Phys. Rev. B* **30**, 4314–4321 (1984).

71. M. B. Salamon, M. E. Meichle, Premartensitic phases of $Ti_{50}Ni_{47}Fe_3$. *Phys. Rev. B Condens Matter.* **31**, 7306–7315 (1985).

72. G. R. Barsch, J. A. Krumahansl, Nonlinear and nonlocal continuum model of transformation precursors in martensites. *Metall. Trans. A* **19**, 761–775 (1988).

73. F. E. Fujita, *Physics of New Materials* (Springer, 1998).

74. Y. Kuroiwa, H. Muramoto, T. Shobu, H. Tokumichi, Y. Noda, Y. Yamada, Pretransitional phenomena at the first-order phase transition in $LaNbO_4$. *J. Phys. Soc. Jpn.* **64**, 3798–3803 (1995).

75. J. M. Kiat, G. Calvarin, Y. Yamada, Anomalous incommensurability and embryonic fluctuations in lead phosphate $Pb_3(PO_4)_2$. *Phys. Rev. B* **48**, 34–41 (1993).

76. G. Burns, F. H. Dacol, Polarization in the cubic phase of $BaTiO_3$. *Solid State Commun.* **42**, 9–12 (1982).

77. R. Z. Tai, K. Namikawa, A. Sawada, M. Kishimoto, M. Tanaka, P. Lu, K. Nagashima, H. Maruyama, M. Ando, Picosecond view of microscopic-scale polarization clusters in paraelectric $BaTiO_3$. *Phys. Rev. Lett.* **93**, 087601 (2004).

78. A. ZięBińska, D. Rytz, K. Szot, M. G'orny, K. Roleder, Birefringence above $T_C$ in single crystals of barium titanate. *J. Phys. Condens. Matter* **20**, 142202 (2008).

79. E. Dul'kin, J. Petzelt, S. Kamba, E. Mojaev, M. Roth, Relaxor-like behavior of $BaTiO_3$ crystals from acoustic emission study. *Appl. Phys. Lett.* **97**, 032903 (2010).

80. S. Tsukada, Y. Fujii, S. Kojima, Y. Akishige, Angle-resolved polarized Raman scattering from $BaTiO_3$ crystals. *Proc. Symp. Ultra. Electron.* **36**, 3J3–3 (2015).

81. V. Mishra, A. Sagdeo, V. Kumar, M. K. Warshi, H. M. Rai, S. K. Saxena, D. R. Roy, V. Mishra, R. Kumar, P. R. Sagdeo, Electronic and optical properties of $BaTiO_3$ across tetragonal to cubic phase transition: An experimental and theoretical investigation. *J. Appl. Phys.* **122**, 065105 (2017).

82. M. Paściak, S. E. Boulfelfel, S. Leoni, Polarized cluster dynamics at the paraelectric to ferroelectric phase transition in $BaTiO_3$. *J. Phys. Chem. B* **114**, 16465–16470 (2010).

83. B. A. Frandsen, L. Liu, S. C. Cheung, Z. Guguchia, R. Khasanov, E. Morenzoni, T. J. S. Munsie, A. M. Hallas, M. N. Wilson, Y. Cai, G. M. Luke, B. Chen, W. Li, C. Jin, C. Ding, S. Guo, F. Ning, T. U. Ito, W. Higemoto, S. J. L. Billinge, S. Sakamoto, A. Fujimori, T. Murakami, H. Kageyama, J. A. Alonso, G. Kotliar, M. Imada, Y. J. Uemura, Volume-wise

destruction of the antiferromagnetic Mott insulating state through quantum tuning. *Nat. Commun.* **7**, 12519 (2016).

84. A. S. Mcleod, E. van Heumen, J. G. Ramirez, S. Wang, T. Saerbeck, S. Guenon, M. Goldflam, L. Anderegg, P. Kelly, A. Mueller, M. K. Liu, I. K. Schuller, D. N. Basov, Nanotextured phase coexistence in the correlated insulator $V_2O_3$. *Nat. Phys.* **13**, 80–86 (2017).

85. D. B. McWhan, M. Marezio, J. P. Remeika, P. D. Dernier, X-ray diffraction study of metallic $VO_2$. *Phys. Rev. B* **10**, 490–495 (1974).

86. A. Bianconi, S. Stizza, R. Bernardini, Critical behavior of the plasmon resonance at the metal-insulator transition in $VO_2$. *Phys. Rev. B* **24**, 4406–4411 (1981).

87. M. M. Qazilbash, M. Brehm, B.-G. Chae, P.-C. Ho, G. O. Andreev, B.-J. Kim, S. J. Yun, A. V. Balatsky, M. B. Maple, F. Keilmann, H.-T. Kim, D. N. Basov, Mott transition in $VO_2$ revealed by infrared spectroscopy and nano-imaging. *Science* **318**, 1750–1753 (2007).

88. J. Laverock, S. Kittiwatanakul, A. A. Zakharov, Y. R. Niu, B. Chen, S. A. Wolf, J. W. Lu, K. E. Smith, Direct observation of decoupled structural and electronic transitions and an ambient pressure monocliniclike metallic phase of $VO_2$. *Phys. Rev. Lett.* **113**, 216402 (2014).

89. T. Katsufuji, T. Kajita, S. Yano, Y. Katayama, K. Ueno, Nucleation and growth of orbital ordering. *Nat. Commun.* **11**, 2324 (2020).

90. H. Soo, M. Krüger, Fluctuational electrodynamics for nonlinear materials in and out of thermal equilibrium. *Phys. Rev. B* **97**, 045412 (2018).

91. K. Asheichyk, Time-dependent radiative heat flux after the beginning of thermal radiation. *Phys. Rev. B* **111**, 075408 (2025).

92. S. Yang, M. Liu, C. Zhao, S. Fan, C.-W. Qiu, Nonreciprocal thermal photonics. *Nat. Photonics* **18**, 412–424 (2024).

93. V. Petrícek, M. Dušek, L. Palatinus, Crystallographic computing system JANA2006: General features. *Z. Kristallogr. Cryst. Mater.* **229**, 345–352 (2014).

94. P. E. Blöchl, Projector augmented-wave method. *Phys. Rev. B* **50**, 17953 (1994).

95. J. P. Perdew, A. Ruzsinszky, G. I. Csonka, O. A. Vydrov, G. E. Scuseria, L. A. Constantin, X. Zhou, K. Burke, Restoring the density-gradient expansion for exchange in solids and surfaces. *Phys. Rev. Lett.* **100**, 136406 (2008).

96. P. Giannozzi, S. Baroni, N. Bonini, M. Calandra, R. Car, C. Cavazzoni, D. Ceresoli, G. L. Chiarotti, M. Cococcioni, I. Dabo, QUANTUM ESPRESSO: A modular and open-source software project for quantum simulations of materials. *J. Phys. Condens. Matter* **21**, 395502 (2009).

97. T. Tadano, Y. Gohda, Tsuneyuki, Anharmonic force constants extracted from first-principles molecular dynamics: Applications to heat transfer simulations. *J. Phys. Condens. Matter* **26**, 225402 (2014).

98. Y. Oba, T. Tadano, R. Akashi, S. Tsuneyuki, First-principles study of phonon anharmonicity and negative thermal expansion in $ScF_3$. *Phys. Rev. Mater.* **3**, 033601 (2019).

99. G. Kresse, J. Furthmuller, Efficient iterative schemes for ab initio total-energy calculations using a plane-wave basis set. *Phys. Rev. B* **54**, 11169–11186 (1996).

100. R. O. Bell, G. Rupprecht, Elastic constants of strontium titanate. *Phys. Rev.* **129**, 90–94 (1963).

101. R. J. Schiltz Jr., J. F. Smith, Elastic constants of some $M\mathrm{Al}_2$ single crystals. *J. Appl. Phys.* **45**, 4681–4685 (1974).

102. N. Nakanishi, Elastic constants as they relate to lattice properties and martensite formation. *Prog. Mater. Sci.* **24**, 143–265 (1980).

103. M. Glogarova, Elastic anomalies in the paraelectric phases of $Tl_2Cd_2(SO_4)_3$ and $(NH_4)_2Cd_2(SO_4)_3$. *Phys. Stat. Sol. A* **22**, K69–K71 (1974).

104. Y. Luspin, G. Hauret, Study of the velocity and damping of acoustic waves obtained by Brillouin scattering in the paraelastic phase in GMO. *Phys. Stat. Sol. B* **76**, 551–558 (1976).

105. R. D. Shannon, Revised effective ionic radii and systematic studies of interatomic distances in halides and chalcogenides. *Acta Crystallogr. Sect. A* **32**, 751–767 (1976).

106. R. W. Boyd, *Nonlinear Optics (Fourth Edition*) (Academic Press, 2020).

107. T. Cao, K. Liu, Y. Tang, J. Deng, K. Li, G. Li, A high-index $Ge_2Sb_2Te_5$-based Fabry–Perot cavity and its application for third-harmonic generation. *Laser Photonics Rev.* **13**, 1900063 (2019).

108. M. Mączka, A. Nowok, J. K. Daręba, D. Stefańska, A. Gągor, M. Trzebiatowska, A. Sieradzki, Near-infrared phosphorescent hybrid organic-inorganic perovskite with high-contrast dielectric and third-order nonlinear optical switching functionalities. *ACS Appl. Mater. Interfaces* **14**, 1460 (2022).

109. C. Dues, M. J. Müller, S. Chatterjee, C. Attaccalite, S. Sanna, Nonlinear optical response of ferroelectric oxides: First-principles calculations within the time and frequency domains. *Phys. Rev. Mater.* **6**, 065202 (2022).

110. D. A. Litvinov, O. M. Kushchenko, P. I. Lazarenko, A. O. Yakubov, A. V. Yulin, A. D. Sinelnik, High-efficient switchable third harmonic generation on thin film phase change materials. *Laser Photonics Rev.* **2025**, e01669 (2025).

111. K. Samanta, S. Dussan, R. S. Katiyar, P. Bhattacharya, Structural and optical properties of nanocrystalline $Zn_{1-x}Mn_xO$. *Appl. Phys. Lett.* **90**, 261903 (2007).

112. M. Balkanski, R. F. Wallis, E. Haro, Anharmonic effects in light scattering due to optical phonons in silicon. *Phys. Rev. B* **28**, 1928 (1983).

113. P. Kumar, P. Lemmens, M. K. Ghosh, F. Ludwig, M. Schilling, Effect of HF concentration on physical and electronic properties of electrochemically formed nanoporous silicon. *J. Nanomater.* **2009**, 728957 (2009).

114. S. Osswald, V. N. Mochalin, M. Havel, G. Yushin, Y. Gogotsi, Phonon confinement effects in the Raman spectrum of nanodiamond. *Phys. Rev. B* **80**, 075419 (2009).

115. M. Uehara, S. Mori, C. H. Chen, S.-W. Cheong, Percolative phase separation underlies colossal magnetoresistance in mixed-valent manganites. *Nature* **399**, 560–563 (1999).

116. A. Moreo, S. Yunoki, E. Dagotto, Phase separation scenario for manganese oxides and related materials. *Science* **283**, 2034–2040 (1999).

117. D. Fu, H. Taniguchi, M. Itoh, S. Y. Koshihara, N. Yamamoto, S. Mori, Relaxor $Pb(Mg_{1/3}Nb_{2/3})O_3$: A ferroelectric with multiple inhomogeneities. *Phys. Rev. Lett.* **103**, 207601 (2009).

118. F. Chu, N. Setter, A. K. Tagantsev, The spontaneous relaxor-ferroelectric transition of $Pb(Sc_{0.5}Ta_{0.5})O_3$. *J. Appl. Phys.* **74**, 5129–5134 (1993).

119. Y. Moritomo, A. Machida, T. Nonobe, K. Ohoyama, Neutron investigation of Ru-doped $Nd_{1/2}Ca_{1/2}MnO_3$ –Comparison with Cr-doped $Nd_{1/2}Ca_{1/2}MnO_{3-}$. *J. Phys. Soc. Jpn.* **71**, 1626–1629 (2002).

120. F. Zhou, W. Nielson, Y. Xia, V. Ozoliņš, Lattice anharmonicity and thermal conductivity from compressive sensing of first-principles calculations. *Phys. Rev. Lett.* **113**, 185501 (2014).

121. A. Togo, First-principles phonon calculations with phonopy and phono3py. *J. Phys. Soc. Jpn.* **92**, 012001 (2023).

122. A. Togo, L. Chaput, T. Tadano, I. Tanaka, G. Hug, Implementation strategies in phonopy and phono3py. *J. Phys. Condens. Matter.* **35**, 353001 (2023).

123. G. Kresse, D. Joubert, From ultrasoft pseudopotentials to the projector augmented-wave method. *Phys. Rev. B* **59**, 1758–1775 (1999).
